# An Alkaloid and a Steroid from the Endophytic Fungus *Aspergillus fumigatus*

**DOI:** 10.3390/molecules20011424

**Published:** 2015-01-14

**Authors:** Zizhen Liang, Tiantian Zhang, Xiaoqian Zhang, Jia Zhang, Changqi Zhao

**Affiliations:** Key Laboratory of Cell Proliferation and Regulation Biology, Ministry of Education, College of Life Science, Beijing Normal University, Beijing 100875, China; E-Mails: liangzizhen008@163.com (L.Z.); bnuzhangtiantian@163.com (T.Z.); zhangxiaobei42@163.com (X.Z.); zhangjia047@163.com (J.Z.)

**Keywords:** *Diphylleia sinensis* L, *Aspergillus fumigates*, indolediketopiperazine alkaloids, steroid, cytotoxicity

## Abstract

Two new compounds, fumitremorgin 12-methoxy-13-[5'-hydroxy-2'-(1''-hydroxy-3''-methoxy-5''-methylbenzoyl)-3'-methoxy]benzoic acid methyl ester (fumitremorgin D, **1**) and 4,8,10,14-tetramethyl-6-acetoxy-14-[16-acetoxy-19-(20,21-dimethyl)-18-ene]-phenanthrene-1-ene-3,7-dione (**2**) were isolated from the cultured endophytic isolated fungus *Aspergillus fumigatus*, together with fourteen known compounds. Their structures were elucidated by 1-D and 2-D NMR analyses. The cytotoxicity profile of the compound against the human hepatocellular carcinoma cell line HepG2 was evaluated by MTT antiproliferative assays.

## 1. Introduction

Many microorganism-originated secondary metabolites have been utilized as drugs and/or lead compounds in the pharmaceutical industry [1,2]. The specific metabolic pathways, habitats and bioactivities of endophytic fungi make them a good source of structurally novel and/or biologically active secondary metabolites [3,4]. Fungi of the genus *Aspergillus* (Moniliaceae) have been reported as prolific producers of bioactive compounds [5,6,7,8]. In the course of our investigation of endophytic fungi harbored in plant tissues, the fungus Wrq12 was isolated from *Diphylleia sinensis*. L and identified as *Aspergillus fumigatus*. *Diphylleia sinensis*. L (also called “WO-ER-CHI” in Traditional Chinese Medicine), is mainly distributed in the midwest of China, and is generally used for the treatment of rheumatic arthritis, lumbocrural pain, traumatic injury, irregular menstruation, etc. [9]. Further fermentation and fractionation of the chloroform extract of *A. fumigatus* mediums led to the isolation of two new compounds **1**–**2** along with the fourteen known compounds fumitremorgin C (**3**) [10], 12,13-dihydroxyfumitremorgin C (**4**) [11], verruculogen (**5**) [12], 13-oxoverruculogen (**6**) [7], ergosteryl peroxide (**7**) [13], helvolic acid (**8**) [14], emodin 1,6-dimethyl ether (**9**) [15], isorhodoptilometrin (**10**) [16], monomethylsulochrin (**11**) [17], trypacidin (**12**) [18], fumigaclavine C (**13**) [19], fumigaclavine A (**14**) [19], fumiquinazoline C (**15**) [20] and pseurotin A (**16**) [21]. The structures of the compounds were established on the basis of spectroscopic analyses and by comparison of their data with literature values.

## 2. Results and Discussion

Compound **1** was obtained as a yellow amorphous powder. The IR spectroscopic data indicated the presence of ether groups (1107 cm^−1^ and 1241 cm^−1^), amide groups (1631 cm^−1^), an amine group (3431 cm^−1^) and an ester group (1723 cm^−1^). Its molecular formula was determined as C_40_H_41_N_3_O_11_ by HR-ESIMS (*m/z* 740.2806 [M+H]^+^, C_40_H_42_N_3_O_11_^+^ calc. 740.2814), requiring 22 sites of unsaturation for the whole molecule. All 40 carbons and 38 of 41 protons can be identified in the ^13^C- and ^1^H-NMR spectra of compound **1**, and HSQC correlations suggest the presence of three exchangeable protons. Salient features of the molecule including seven methyl singlets, three sp^3^-hybridized methylenes, three sp^3^-hybridized and eight sp^2^-hybridized methines, one sp^3^-hybridized and fourteen sp^2^-hybridized quaternaries, one ketone (δ 199.9) and three ester or amide carbonyls were indicated by the NMR data.

**Figure 1:**
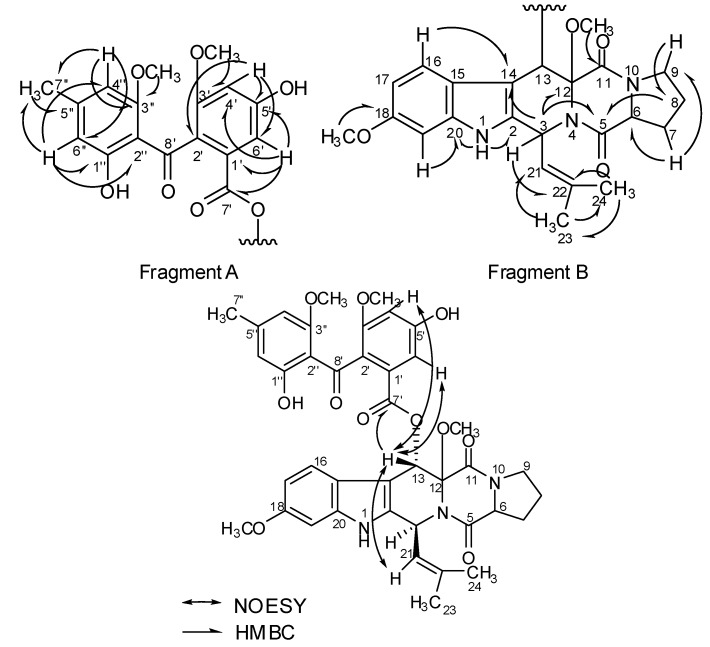
Key HMBC and NOESY correlations for compound **1**.

By comparison with the literature data [11], fragment B in compound **1** was most likely an indole-diketopiperazine skeleton (Figure 1), which is indicated by the carbon combination, along with the presence of an amide group and amine group. This deduction was further confirmed by a correlative interpretation of its NOESY and HMBC spectrum (Table 1). Although no direct HMBC correlation between the methoxyl group (δ 3.62 (3H, s), δ 52.1) and C-12 (δ 83.1) was observed, the chemical shift of C-12 at δ 83.1 indicated that the methoxyl group was linked at C-12.

The remaining signals of compound **1** were identified by the HMBC data (Figure 1) and comparisons with the literature data [17]. The HMBC correlations between δ 6.06 (s, H-4'') and carbon resonance at δ 22.4 (7''-CH_3_), δ 110.9 (C-6'') and δ 161.0 (C-3'') together with the correlations between δ 6.45 (s, H-6'') and carbon resonance at δ 22.4 (7''-CH_3_), δ 103.0 (C-4''), δ 110.5 (C-2'') and δ 164.0 (C-1'') suggested a 1'', 2'', 3'' and 5''-tetrasubstituted aromatic ring. Likewise, another 1', 2', 3' and 5'-tetrasubstituted aromatic ring was identified by the HMBC correlations. The structure of fragment A was confirmed as rhizoctonic acid. It can directly be located at C-13 by key HMBC correlation from δ 5.75 (H-13) to δ 166.2 (C-7'). This linkage was also supported by the key NOESY correlation (Figure S10–S11 Supplementary Files) between H-13 (δ 5.75) and both H-4' (δ 6.59) and H-6' (δ 7.00). The observation of a NOESY correlation between H-13 (δ 5.75) and H-21 (δ 4.78) showed H-13 was *cis* to the 2-methylprop-1-ene moiety and *trans* to H-3 as shown (Figure 1). Thus, the gross structure of compound 1 was determined as 12-methoxy-13-[5'-hydroxy-2'-(1''-hydroxy-3''-methoxy-5''-methyl-benzoyl)-3'-methoxy]benzoic acid methyl ester-fumitremorgin, which was named fumitremorgin D.

**Table 1:** NMR Spectroscopic Data (400 MHz, CDCl_3_) for compound **1**.

fumitremorgin D (1)						
Position		δ_C_, type		δ_H_ ( *J* in Hz)	HMBC	NOESY
1-NH				8.02, s	2, 14, 15, 20	
2		130.1, C				
3		50.2, CH		5.82, d (9.5)	2, 5, 12, 14, 21, 22,	H-16, H-21, H-24
4						
5		171.1, C				
6		58.8, CH		4.45, dd (8.0, 12.0)	5, 7	H-7, H-8
7		29.1, CH_2_		2.06, 2.45, m	6, 8, 9	H-6, H-8
8		22.5, CH_2_		1.94, 2.08, m	5	H-6, H-9
9		45.3, CH_2_		3.64, d (8.0)	7, 8	H-7
10						
11		166.4, C				
12		83.1, C				
13		68.7, CH		5.75, s	2, 12, 14, 7'	H-16, H-21, H-4', H-6'
14		105.2, C				
15		120.7, C				
16		121.1, CH		7.78, d (8.0)	14, 18, 20	H-13, H-19
17		109.8, CH		6.78, d (8.0)	15, 19	18-OCH_3_, H-16
18		156.6, C				
19		95.1, CH		6.80, s	15, 17, 18, 20	18-OCH_3_, H-16
20		137.6, C				
17		109.8, CH		6.78, d (8.0)	15, 19	18-OCH_3_, H-16
21		123.8, CH		4.78, d (9.3)	23, 24	H-3, H-23
22		134.7, C				
23		25.6, CH_3_		1.64, s	21, 22, 24	H-12, H-24
24		18.2, CH_3_		1.96, s	21, 22, 23	H-23
1'		128.4, C				
2'		127.5, C				
3'		157.0, C				
4'		103.4, CH		6.59, s	108.1; 127.5; 156.9	H-13, 3'-OCH_3_
5'		156.9, C				
6'		108.1, CH		7.00, s	103.4; 127.5; 156.9; 166.2	H-13
7'		166.2, C				
8'		199.9, C				
1''		164.0, C				
2''		110.5, C				
3''		161.0, C				
4''		103.0, CH		6.06, s	8', 3'', 6'', 7''	H-7'', 3''-OCH_3_
5''		148.0, C				
6''		110.9, CH		6.45, s	1'', 2'',4'', 7''	H-7''
7''		22.4, CH_3_		2.28, s	4'', 5''	H-4'', H-6''
C1''-OH				12.99, s	1'', 5'', 6''	H-3''-OCH_3_
18-OCH_3_		55.7, CH_3_		3.81, s	18	H-19
3''-OCH_3_		55.7, CH_3_		3.36, s	13''	H-4''
3'-OCH_3_		56.1, CH_3_		3.63, s	3'	H-4'
12-OCH_3_		52.1, CH_3_		3.62, s	11	H-8

Compound **2** was isolated as an amorphous solid. Its molecular formula was determined as C_32_H_46_O_6_ by HR-ESI(+)MS [M+Na]^+^ *m/z* 509.2878 (calcd for C_29_H_42_NaO_6_^+^, 509.2874), requiring nine sites of unsaturation. The ^1^H- and ^13^C-NMR data (Table 2) revealed 27 carbon resonance lines and all 42 protons. Scrutiny of its ^1^H- and ^13^C-NMR data, in correlation with DEPT and HSQC experiments, indicated the ^13^C resonances of 14-CH_3_ and 23-CH_3_ overlap (δ 17.5). The ^13^C resonances appearing in δ 40.5 (C-15), was covered by the DMSO peaks. Thus, the ^1^H, ^13^C, DEPT and HSQC NMR data for compound **2** revealed the presence of eight methyl singlets, five sp^3^-hybridized methylenes, five sp^3^-hybridized and three sp^2^-hybridized methines, three sp^3^-hybridized and one sp^2^-hybridized quaternaries, two ketones (δ 200.8 and 209.3) and two ester carbons. These carbon combinations indicated that compound **2** was most likely a helvolic acid derivative [14].

**Table 2:** NMR data for compound **2** in DMSO-*d*_6_.

4,8,10,14-tetramethyl-6-acetoxy-14-[16-acetoxy-19-(20,21-dimethyl)-18-ene]-phenanthrene-1-ene-3,7-dione (2)					
Position	δ_C_, type	δ_H_ ( *J* in Hz)	HMBC	COSY	NOESY
1	158.4, CH	7.42, d (10.0)	3, 5, 10		
2	126.8, CH	5.76, d (10.0)	10	H-1	
3	200.8, C				
4	39.5, CH	2.74, m	4-CH_3_		
4-CH_3_	12.3, CH_3_	1.12, d (7.2)	3, 4, 5	H-4	H-6
5	45.6, CH	2.41, m	4	H-4	H-6, H-8-CH_3_
6	73.0, CH	5.04, s	7, 8, 10, 6-OAc		H-5, H-4-CH_3_
6-OAc	169.0, C				
CH_3_	20.4, CH_3_	2.09, s	6-OAc		
7	209.3, C				
8	52.3, CH				
8-CH_3_	17.5, CH_3_	1.10, s	7, 8, 9, 14		H-5, H-15
9	41.2, CH	2.54, m			
10	37.7, C				
10-CH_3_	27.1, CH_3_	1.39, s	1, 5, 9, 10		H-4
11	23.3, CH_2_	1.57, 1.87, m	12		
12	25.5, CH_2_	1.70, 2.29, m		H-13	
13	29.0, CH_2_	2.31, 1.22, m		H-12	
14	46.1, C				
14-CH_3_	17.5, CH_3_	0.81, s	8, 14, 15		
15	40.5, CH_2_	1.64, 2.05, d (8.4)	13, 16, 14-CH_3_		
16	73.3, CH	5.70, br	14	H-17	
16-OAc	169.9, C				
CH_3_	20.5, CH_3_	1.87, s	16-OAc		
17	28.1, CH_2_	1.99; 2.09, m		H-16	
18	124.1, CH	5.11, t (8.0)		H-17	
19	130.8, C				
20	17.5, CH_3_	1.58, s	21, 22, 24		
21	20.5, CH_3_	1.65, s	21, 22, 23		

**Figure 2:**
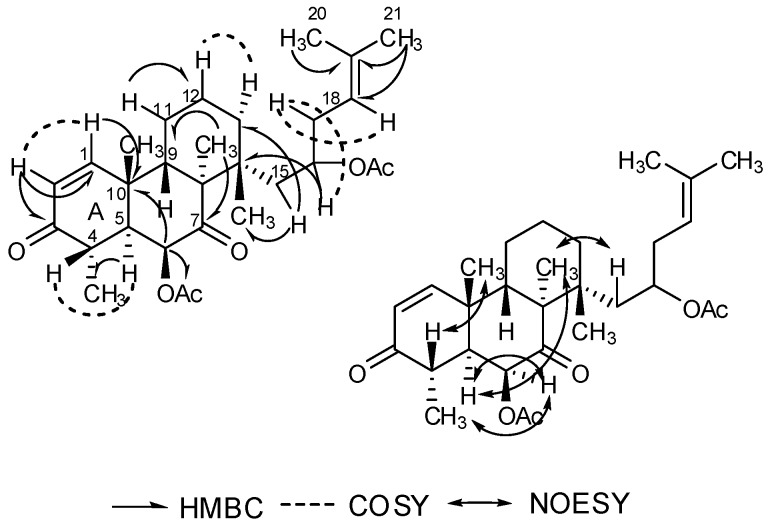
Key NMR correlations for compound 2.

We confirmed the assignment of a tricyclic skeleton (A, B and C ring) by correlative interpretation of its COSY, NOESY, HSQC and HMBC spectroscopic data and comparison with literature [14]. The HMBC data from H-15 (δ 1.64) to 14-CH_3_ (δ 17.5) and from H-16 (δ 5.70) to C-14 (δ 46.1) suggest a side chain connected at the site of C-14 of the skeleton. HMBC correlations from δ 1.58 and δ 1.65 to δ 124.1 and δ 130.8 confirmed a 2-methy-2-yl-butene. The COSY data between H-18 (δ 5.11) and H-17 (δ 2.09), and H-16 (δ 5.70) and H-17 (δ 2.09) indicated the connection between C-16 and C-17 (Figure 2).

The relative configuration of compound 2 was determined by the NOESY correlations and comparison with literature data [14]. The NOESY correlations between H-15 (δ 2.05) and 8-CH_3_ (δ 1.10) showed that the substituent group and 8-CH_3_ were oriented *cis* to each other. Comprehensive analyses of MS and NMR data led to the structural elucidation of compound **2** as 4,8,10,14-tetramethyl-6-acetoxy-14-[16-acetoxy-19-(20,21-dimethyl)-18-ene]-phenanthrene-1-ene-3,7-dione. Structurally, this compound is the first example of a helvolic acid derivative possessing a phenanthrene skeleton.

The cytotoxic activities of all compounds were evaluated by an MTT assay using the HepG2 cell line. Compounds **1** and **2** both showed weak cytotoxicity in this assay, with IC_50_ values of 47.5 μM and 139.9 μM, respectively. It was noteworthy that 12,13-dihydroxyfumitremorgin C (**4**), and verruculogen (**5**) showed cytotoxic activity against the HepG2 cell line with IC_50_ values of 4.5 μM and 9.8 μM, respectively. Despite the lack of a macrocyclic structure, 12,13-dihydroxyfumitremorgin C (**4**) showed improved activity over verruculogen (**5**), suggesting that the macrocyclic linking at 1-N and 3-C does not play a crucial role in the observed cytotoxicity. Meanwhile, compounds **1**, **3** and **6** lacking C-12 and/or C-13 hydroxyls, showed IC_50_ values of 47.5, 156.5 and 44.9 μM, respectively. Based on above results, the simultaneous presence of hydroxyls at C-12 and C-13 showed an important structure-activity relationship (SAR) to the cytotoxic activity of indolediketopiperazine alkaloids against the HepG2 cell line.

## 3. Experimental Section

### 3.1. General Procedures

Chemical shifts are given in δ (ppm) with the residual solvent peak referenced to δ_H_ 7.27 and δ_C_ 77.0 for CDCl_3_ and δ_H_ 3.41, 2.51 and δ_C_ 39.5 for DMSO. Column chromatography: (10–40 μm; Marine Chemical Factory, Qingdao, China); Sephadex LH-20 (Amersham Pharmacia Biotech, Uppsala, Sweden); RP-C18 gel (ODS LiChrosorb RP-18, Merck, Darmstadt, Germany) were used for column chromatography. NMR spectroscopic data: Bruker Avance III 400MHz; δ in ppm with SiMe_4_ as internal standard. MS: Bruker micrOTOF-QII mass spectrometer for HR-ESI; IR spectroscopic data: Nexus 670 (Nicolet, Waltham, MA, USA).

### 3.2. Isolation and Cultivation of the Fungus

All plant tissues (mainly roots or rhizomes) of *D. sinensis* L. which were collected from Honghegu (Shanxi Province, China), were cleaned in running tap water and any visibly diseased or damaged material was eliminated. All tissues were surface disinfected with 75% ethanol for 1 min and thrice in sterile distilled water, then disinfected for 5 min in 0.1% mercuric chloride solution and thrice in sterile distilled water and step 1 was then repeated again. After surface sterilization and removal of epidermis the phloem was cut into 0.5–1 cm fragments and inoculated on potato dextrose agar medium (five pieces each) for 3–10 days at 28 °C. Individual colonies were transferred onto new potato dextrose agar medium for further analysis and maintenance. Based on 16s rDNA sequence analysis, strain Wrq12 was classified as *A. fumigatus*. The strain *A. fumigatus* Wrq12 is deposited in the China General Microbiological Culture Collection Center (CGMCC No. 3785).

### 3.3. Preparative Cultivation and Isolation

Forty 500-mL round-bottomed flasks of rice mediums were inoculated with *A. fumigatus*. The flasks were incubated at 27 °C in a constant temperature incubator for 30 days, and then extracted with CDCl_3_ five times to yield 26.4 g of extract after solvent removal. The extract was then partitioned using CHCl_3_/MeOH in a gradient 10:0 to 0:10 elution silica gel column to yield 15 fractions (Fr. 1-Fr. 17). Fraction 12 was chromatographed using CHCl_3_/MeOH in a gradient (10:0 to 3:7) to yield compounds **13**–**14**. Fraction 10 was fractionated by silica gel column chromatography using CHCl_3_/MeOH in a gradient (10:0 to 0:10) to yield 12 fractions (Fr.10-1-Fr.10-12). Fraction 10-6 was chromatographed over a Sephadex LH-20 column, as eluting solvent CHCl_3_/MeOH (1:1) to afford nine fractions (Fr.10-6-9-1-Fr.10-6-9-9). Fractions were further fractionated by repeated column chromatography on Sephadex LH-20 using CHCl_3_/MeOH (1:1) together with ODS column chromatography using MeOH/H_2_O as eluting solvent to afford compounds **1**, **2**, **4**, **8** and **10**. Fraction 10-5 was fractionated by an ODS column using MeOH/H_2_O in a gradient (1:1 to 8:2) to yield compound **11**. Fraction 10-4 was further fractionated by Sephadex LH-20 chromatography using CHCl_3_/MeOH (1:1) as eluting solvent to afford compounds **5**–**7** and **9**. Fraction 10-2 was fractionated by silica gel column chromatography using CHCl_3_/MeOH in a gradient (9:1 to 7:3) to yield compound **12**. Fraction 10-3 was further fractionated by ODS column chromatography using MeOH/H_2_O in a gradient (1:1 to 9:1) to yield compounds **3** and **15**. Fraction 10-7 was fractionated by silica gel column chromatography using CHCl_3_/MeOH in a gradient (10:0 to 9:1) to yield compound **16**. In TLC tests, all compounds isolated showed spots with the same Rf value and TLC color display as the CDCl_3_ extract, indicating that all compounds were isolated from the raw extract.

### 3.4. Cytotoxic Activity Assays

Cytotoxicity was evaluated by the MTT method using HepG2 cell lines. The cell line was grown in DMEM supplemented with 10% FBS under a humidified atmosphere of 5% CO_2_ and 95% air at 37 °C. Then 100 μL of these cell suspensions at a density of 5 × 10^3^ cell per well was plated in 96-well plates and incubated for 16–18 h under the above condition. Then the test compound solutions (in DMSO) were mixed with culture medium and cells were treated with them at gradient concentrations (12.75 μM, 25 μM, 50 μM, 100 μM, 200 μM). After further incubation under the same condition for 24 h, 10 μL of the MTT solution (5 mg/mL in DMEM medium) was added to each well and incubated for 4 h. The old medium containing MTT was then gently replaced by DMSO and standing 20 min to dissolve formazan crystals. Absorbance was then determined on an IMark (Bio-Rad, Hercules, CA, USA) plate reader at 490 nm.

### 3.5. Analytical Data

*Fumitremorgin 12-methoxy-13-[5'-hydroxy-2'-(1''-hydroxy-3''-methoxy-5''-methylbenzoyl)-3'-methoxy]-benzoic acid methyl ester (fumitremorgin D, **1**)*: Amorphous yellow solid; IR ν_max_ 1107, 1241, 1631, 3431, 1723 cm^−1^; ^1^H-NMR (CDCl_3_, 400 MHz) δ 12.99 (1H, s, C1''-OH), 8.02 (1H, s, H-1) , 7.78 (1H, d, *J* = 8.0, H-16), 7.00 (1H, s, H-6'), 6.80 (1H, s, H-19), 6.78 (1H, d, *J* = 8.0, H-17), 6.59 (1H, s, H-4') , 6.45 (1H, s, H-6''), 6.06 (1H, s, H-4''), 5.82 (1H, d, *J* = 9.5 Hz, H-3), 5.75 (1H, s, H-13), 4.78 (1H, d, *J* = 9.3, H-21), 4.45 (1H, dd, *J* = 8.0, 12.0 Hz, H-6), 3.81 (3H, s, 18-OCH_3_), 3.64 (2H, d, *J* = 8.0, H-9), 3.63 (3H, s, 3'-OCH_3_), 3.62 (3H, s, 12-OCH_3_), 3.36 (3H, s, 3''-OCH_3_), 2.45–2.06 (2H, m, H-7), 2.28 (3H, s, H-7''), 2.08–1.94 (2H, m, H-8), 1.96 (3H, s, H-24), 1.64 (3H, s, H-23); ^13^C-NMR (CDCl_3_, 100 MHz) δ 130.1 (C, C-2), 50.2 (CH, C-3), 171.1 (C, C-5), 58.8 (CH_2_, C-6), 29.1 (CH_2_, C-7), 22.5 (CH_2_, C-8), 45.3 (CH_2_, C-9), 166.4 (C, C-11), 83.1 (C, C-12), 68.7 (CH, C-13), 105.2 (C, C-14), 120.7 (C, C-15), 121.1 (CH, C-16), 109.8 (CH, C-17), 156.6 (C, C-18), 95.1 (CH, C-19), 137.6 (C, C-20), 123.8 (CH, C-21), 134.7 (C, C-22), 25.6 (CH_3_, C-23), 18.2 (CH_3_, C-24), 128.4 (C, C-1'), 127.5 (C, C-2'), 157.0 (C, C-3'), 103.4 (CH, C-4'), 156.9 (C, C-5'), 108.1 (CH, C-6'), 166.2 (C, C-7'), 199.9 (C, C-8'), 164.0 (C, C-1''), 110.5 (C, C-2''), 161.0 (C, C-3''), 103.0 (CH, C-4''), 148.0 (C, C-5''), 110.9 (CH, C-6''), 22.4 (CH_3_, C-7''), 55.7 (CH_3_, 18-OCH_3_), 55.7 (CH_3_, 3''-OCH_3_), 56.1 (CH_3_, 3'-OCH_3_), 52.1 (CH_3_, 12-OCH_3_); HR-ESI(+)MS [M+H]^+^ *m/z* 740.2806 (calcd for C_40_H_42_N_3_O_11_^+^, 740.2814).

*4,8,10,14-tetramethyl-6-acetoxy-14-[16-acetoxy-19-(20,21-dimethyl)-18-ene]-phenanthrene-1-ene-3,7-dione* (**2**): Amorphous white solid; ^1^H-NMR (DMSO-*d*_6_, 400 MHz) δ 7.42 (1H, d, *J* = 10.0 Hz, H-1), 5.76 (1H, d, *J* = 10.0, H-2), 2.74 (1H, m, H-4), 1.12 (3H, d, *J* = 7.2, 4-CH_3_), 2.41 (1H, m, H-5), 5.04 (1H, s, H-6), 2.09 (3H, s, 6-OAc-CH_3_), 1.10 (3H, s, 8-CH_3_), 2.54 (1H, m, H-9), 1.39 (3H, s, 10-CH_3_), 1.57, 1.87 (each 1H, m, H-11), 1.70, 2.29 (2H, m, H-12), 2.31, 1.22 (2H, m, H-13), 0.81 (3H, s, 14-CH_3_), 1.64, 2.05 (each 1H, d, *J* = 8.4, H-15), 5.70 (CH, d, *J* = 8.4, H-16), 1.87 (3H, s, 16-OAc-CH_3_), 1.99, 2.09 (2H, m, H-17), 5.11 (1H, t, *J* = 8.0, H-18), 1.58 (3H, s, H-20), 1.65 (3H, s, H-21); ^13^C-NMR (DMSO-*d*_6_, 100 MHz) δ 158.4 (CH, C-1), 126.8 (CH, C-2), 200.8 (C, C-3), 39.5 (CH, C-4), 12.3 (CH_3_, 4-CH_3_), 45.6 (CH, C-5), 73.0 (CH, C-6), 169.0 (C, 6-OAc), 20.4 (CH_3_, 6-OAc-CH_3_), 209.3 (C, C-7), 52.3 (CH, C-8), 17.5 (CH_3_, 8-CH_3_), 41.2 (CH, C-9), 37.7 (C, C-10), 27.1 (CH_3_, 10-CH_3_), 23.3 (CH_2_, C-11), 25.5 (CH_2_, C-12), 29.0 (CH, C-13), 46.1 (C, C-14), 17.5 (CH_3_, 14-CH_3_), 40.5 (CH_2_, C-15), 73.3 (CH, C-16), 169.9 (C, 16-OAc), 20.5 (CH_3_, 16-OAc-CH_3_), 28.1 (CH_2_, C-17), 124.1 (CH, C-18), 130.8 (C, C-19), 17.5 (CH_3_, C-20), 25.5 (CH_3_, C-21); HR-ESI(+)MS [M+Na]^+^ *m/z* 509.2878 (calcd for C_29_H_4__2_NaO_6_^+^, 509.2874).

## 4. Conclusions

In our investigation, sixteen compounds were isolated from the chloroform extract of rice mediums of the endophytic isolated fungus *A. fumigatus*, including two new compounds 12-methoxyl-13-[5'-hydroxy-2'-(1''-hydroxy-3''-methoxy-5''-methylbenzoyl)-3'-methoxy]benzoic acid methyl ester- fumitremorgin (fumitremorgin D, **1**) and 4,8,10,14-tetramethyl-6-acetoxy-14-[16-acetoxy-19-(20,21-dimethyl)-18-ene]-phenanthrene-1-ene-3,7-dione (**2**). A SAR study of the cytotoxicity of indolediketopiperazine alkaloids against the HepG2 cell line was also discussed.

## Supplementary Materials

Supplementary materials can be accessed at: http://www.mdpi.com/1420-3049/20/01/1424/s1.
