# Supplementary material for: An Alkaloid and a Steroid from the Endophytic Fungus Aspergillus fumigatus
Source: Molecules. 2015 Jan 14;20(1):1424–33. doi: 10.3390/molecules20011424 (PMC6272270; doi:10.3390/molecules20011424)
Supplement: Supplementary file 1 [file molecules-20-01424-s001.pdf]

Supplementary Materials

16S rRNA Gene Sequence

CGAAGGGTCACCTTAGAAAAATAAAGTTGGGTGTCGGCTGGCGCCGGCCGGGCCTACAGAGCAGGTGACAAAGCCCCAT
ACGCTCGAGGACCGGACGCGGTGCCGCCGCTGCCTTTCGGGCCCGTCCCCCGGGAGAGGGGGACGGGGGCCCAACACAC
AAGCCGTGCTTGAGGGCAGCAATGACGCTCGGACAGGCATGCCCCCCGGAATACCAGGGGGCGCAATGTGCGTTCAAAG
ACTCGATGATTCACTGAATTCTGCAATTCACATTACTTATCGCATTTCGCTGCGTTCTTCATCGATGCCGGAACCAAGAGA
TCCGTTGTTGAAAGTTTTAACTGATTACGATAATCAACTCAGACTGCATACTTTCAGAACAGCGTTCATGTTGGGGTCTTC
GGCGGGCGCGGGCCCGGGGGCGCAAGGCCTCCCCGGCGGCCGTCGAAACGGCGGGCCCGCCGAAGCAACAAGGTACGAT
AGACACGGGTGGGAGGTTGGACCCAGAGGGCCCTCACTCGGTAATGATCCTTCCGCAGGTTCACCCTACGGAAG

```
LOCUS       KJ175457            550 bp    DNA     linear   PLN 03-FEB-2014
DEFINITION  Aspergillus fumigatus isolate M1103.2732 18S ribosomal RNA gene,
            partial sequence; internal transcribed spacer 1, 5.8S ribosomal RNA
            gene, and internal transcribed spacer 2, complete sequence; and 28S
            ribosomal RNA gene, partial sequence.
ACCESSION   KJ175457
VERSION     KJ175457.1  GI:576867471
KEYWORDS
SOURCE      Aspergillus fumigatus
ORGANISM    Aspergillus fumigatus
            Eukaryota; Fungi; Dikarya; Ascomycota; Pezizomycotina;
            Eurotiomycetes; Eurotiomycetidae; Eurotiales; Aspergillaceae;
            Aspergillus.
REFERENCE   1  (bases 1 to 550)
  AUTHORS   De Respinis,S., Weissenhorn,S., Bosshard,P.P., Petrini,L.E.,
            Tonolla,M. and Petrini,O.
  TITLE     Identification of Aspergillus species in the Flavi and Fumigati
            Sections by matrix-assisted laser desorption/ionization
            time-of-flight mass spectrometry
  JOURNAL   Unpublished
REFERENCE   2  (bases 1 to 550)
  AUTHORS   De Respinis,S., Weissenhorn,S., Bosshard,P.P., Petrini,L.E.,
            Tonolla,M. and Petrini,O.
  TITLE     Direct Submission
  JOURNAL   Submitted (23-JAN-2014) Laboratory of Applied Microbiology,
            University of Applied Sciences of Southern Switzerland (SUPSI), Via
            Mirasole 22A, Bellinzona, Ticino 6501, Switzerland
```

All Compounds Structures

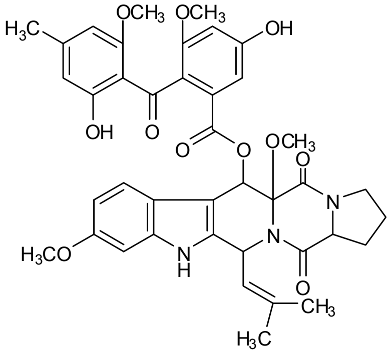

Compound 1

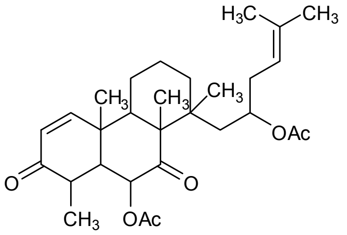

Compound 2

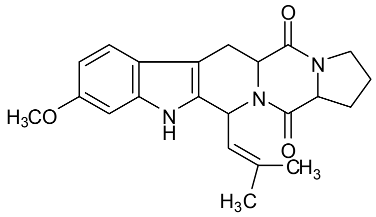

Compound 3

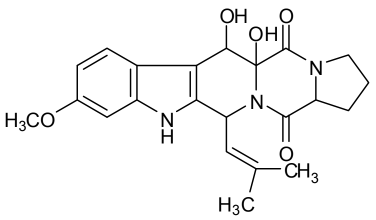

Compound 4

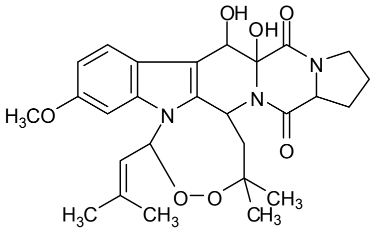

Compound 5

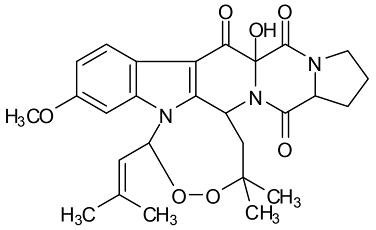

Compound 6

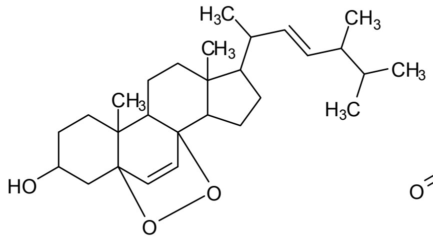

Compound 7

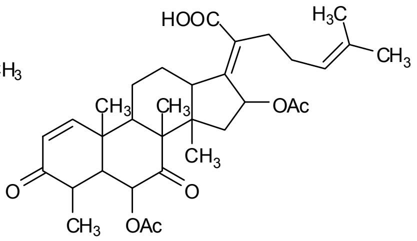

Compound 8

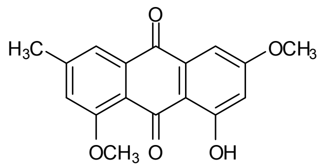

Compound 9

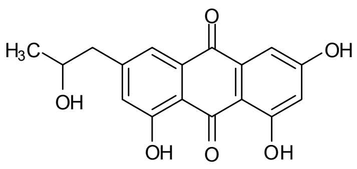

Compound 10

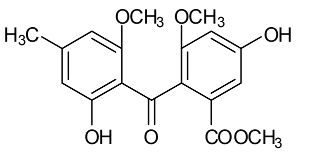

Compound 11

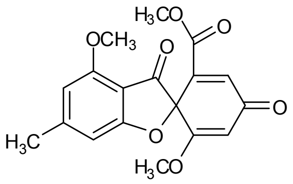

Compound 12

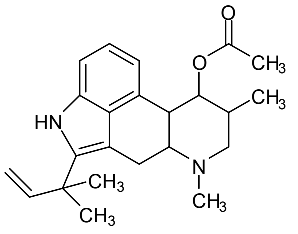

Compound 13

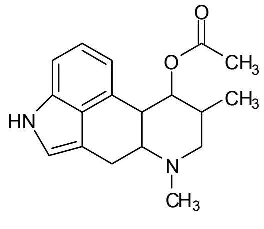

Compound 14

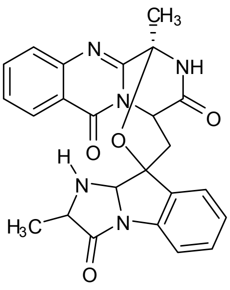

Compound 15

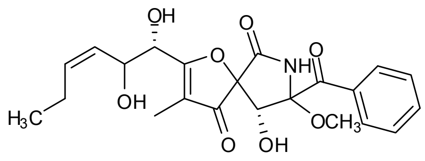

Compound 16

Chart 1. Structure of Compounds **1–16**.

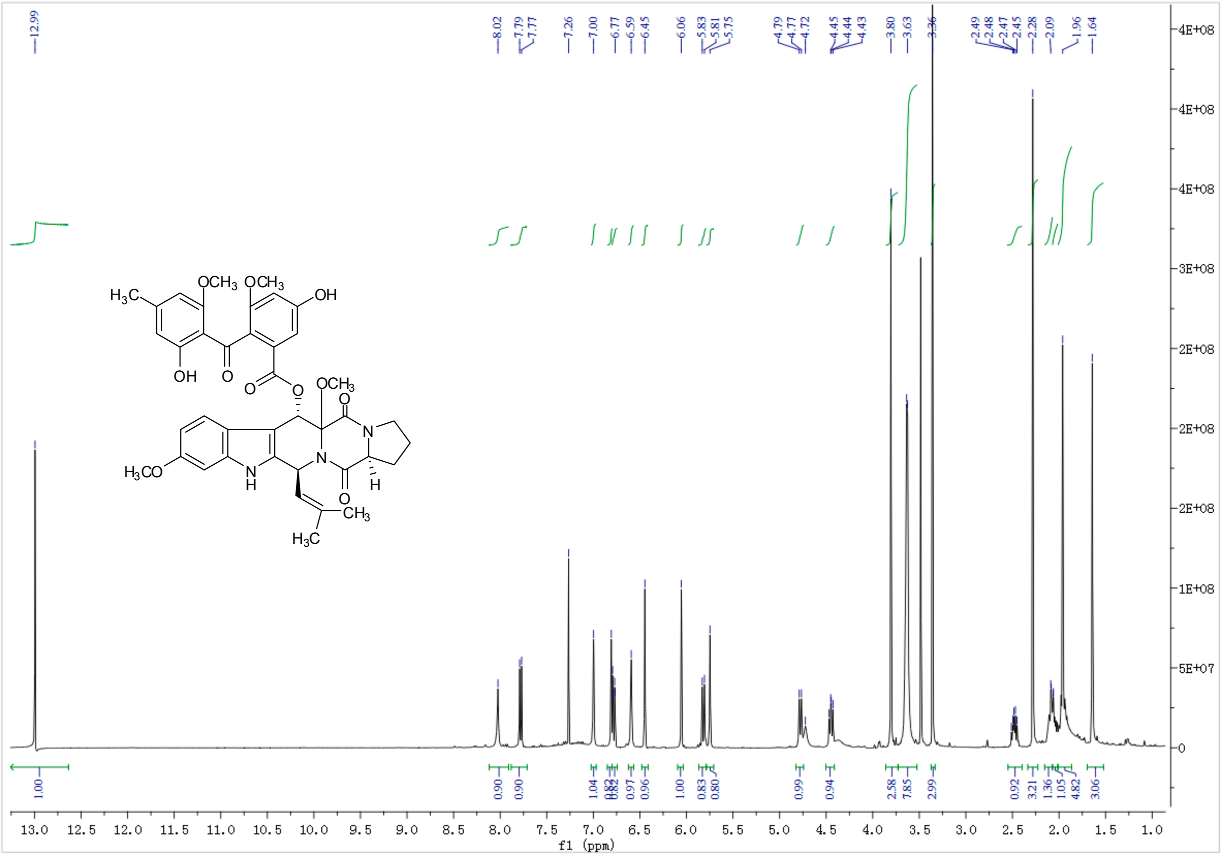

Figure S1. ^{1}H-NMR (400 MHz, $CDCl_3$) spectrum of compound **1**.

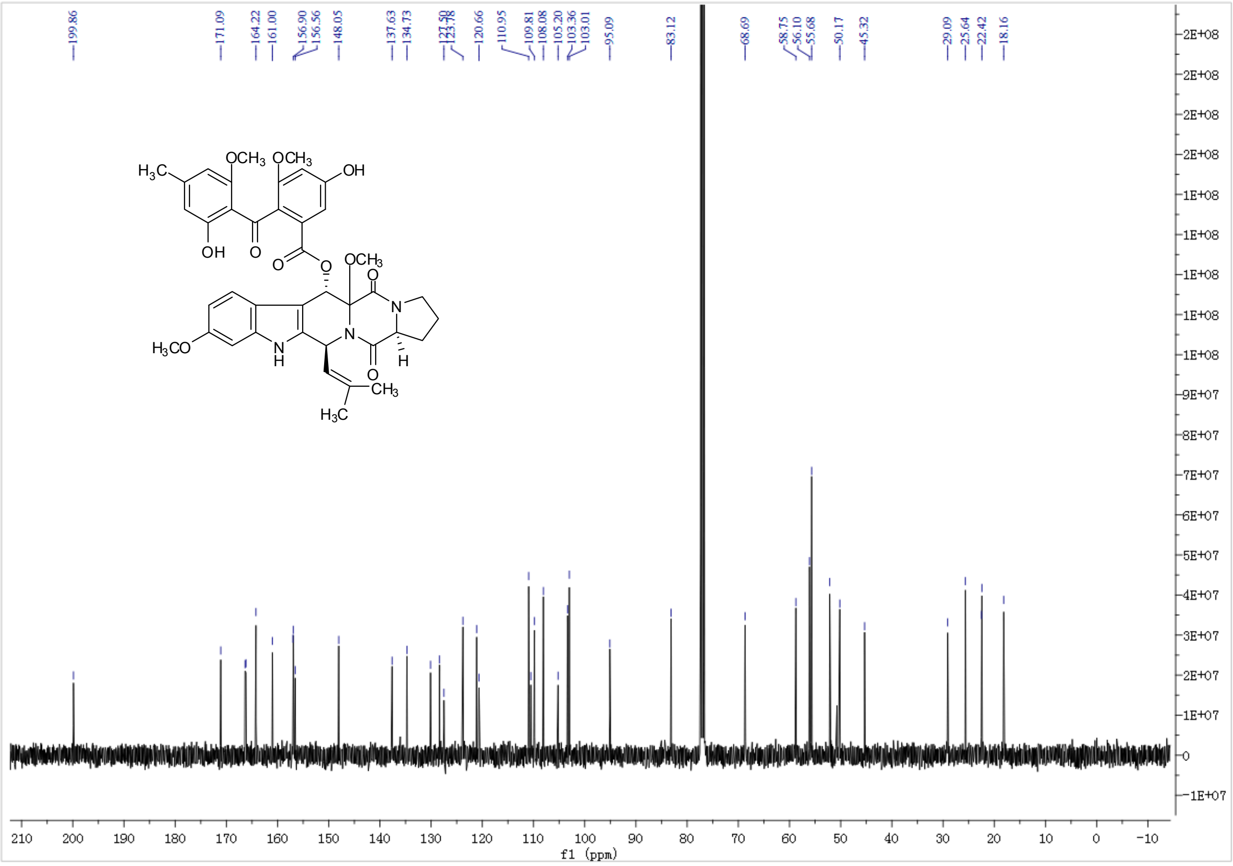

Figure S2. ^{13}C-NMR (100 MHz, $CDCl_3$) spectrum of compound **1**.

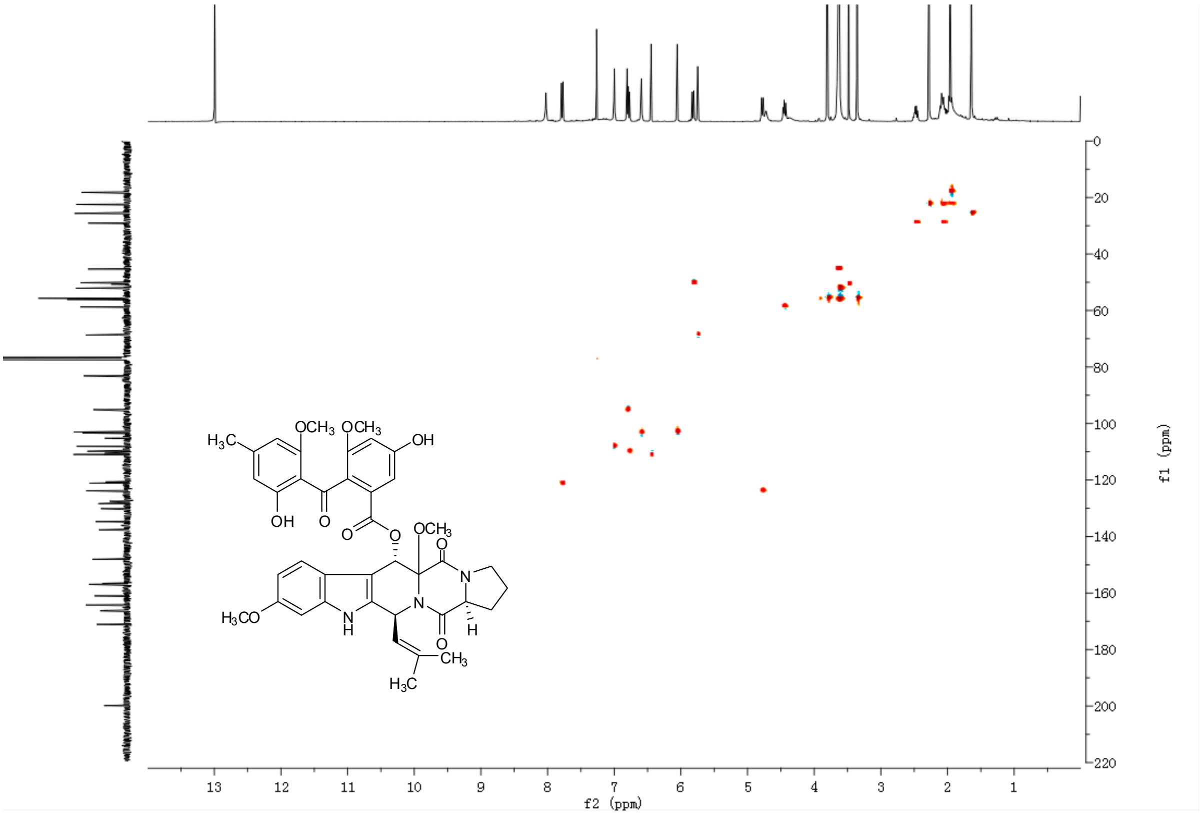

Figure S3. HSQC spectrum of compound **1**.

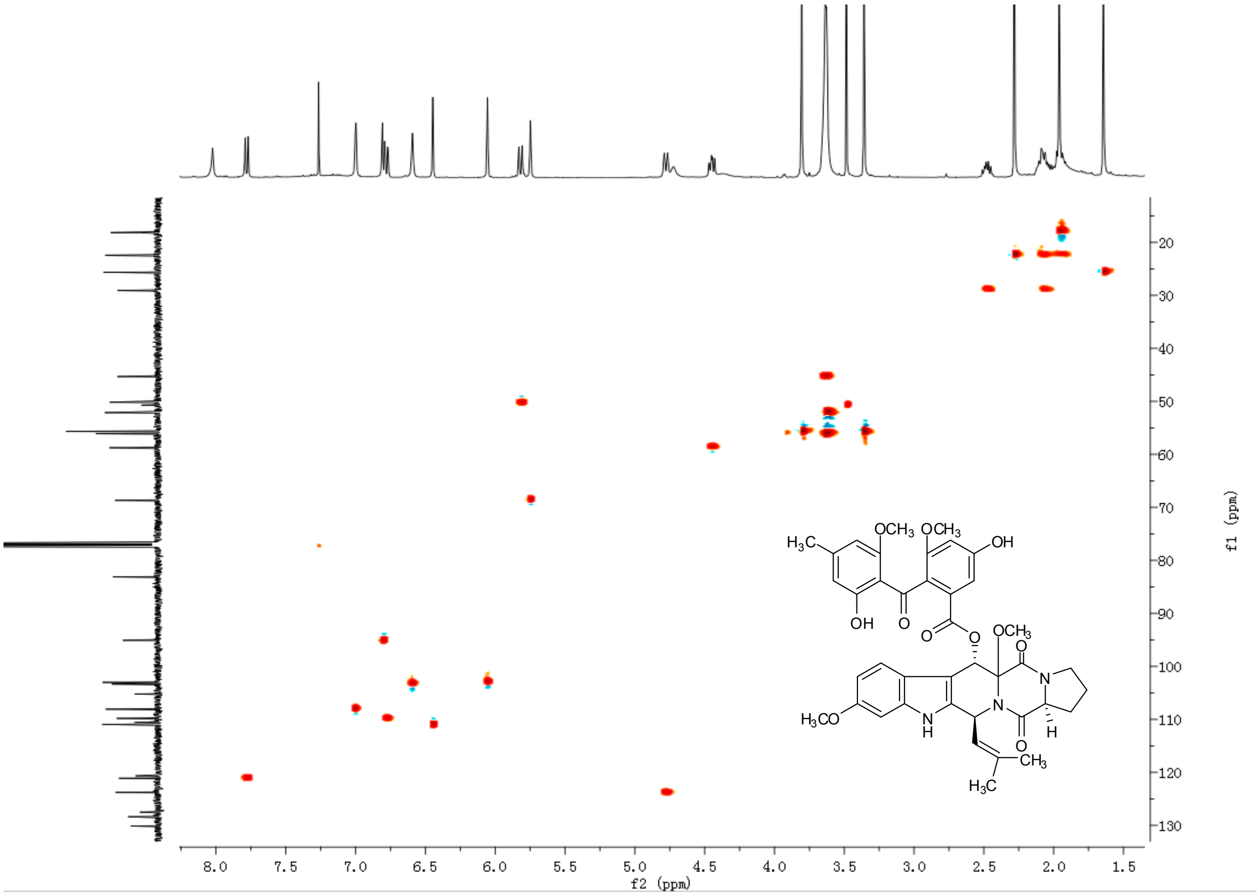

Figure S4. Expanded HSQC spectrum of compound **1**.

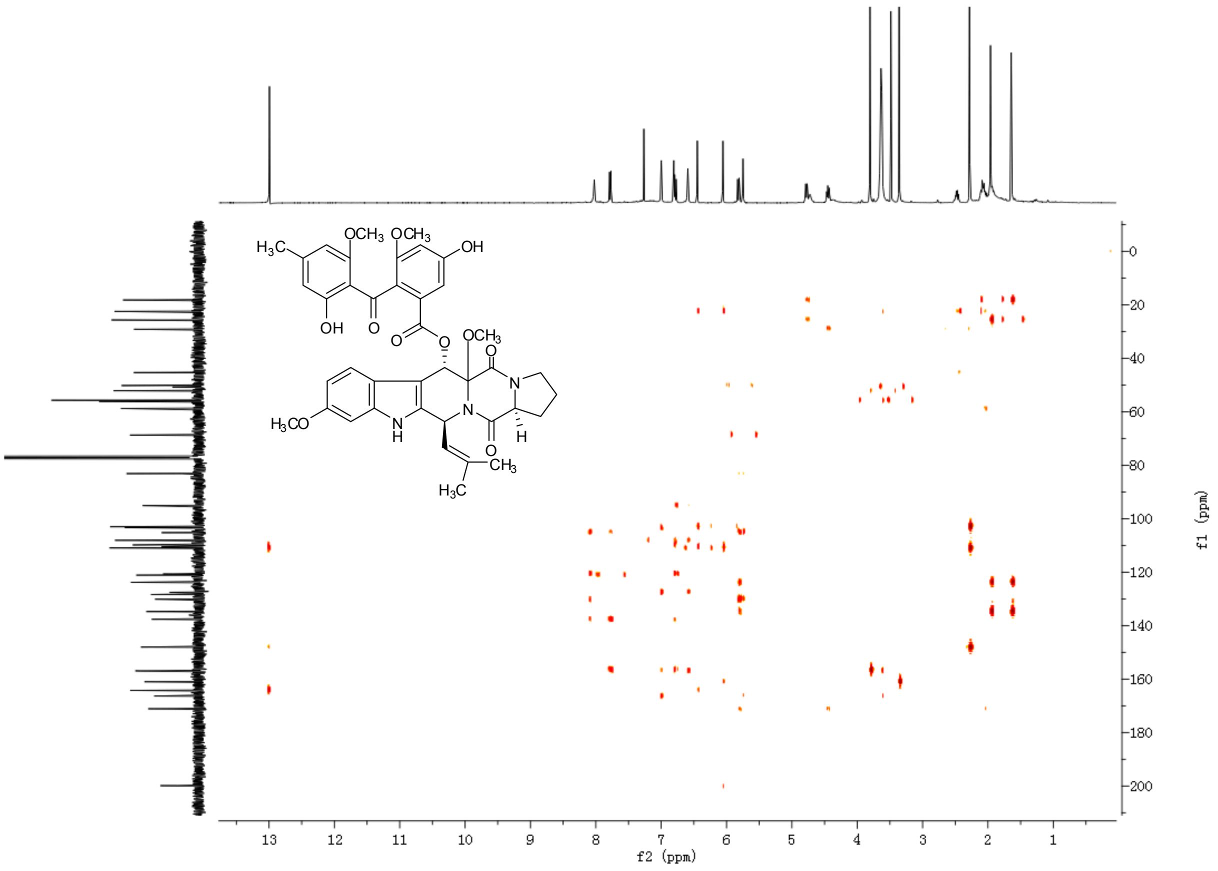

Figure S5. HMBC spectrum of compound **1**.

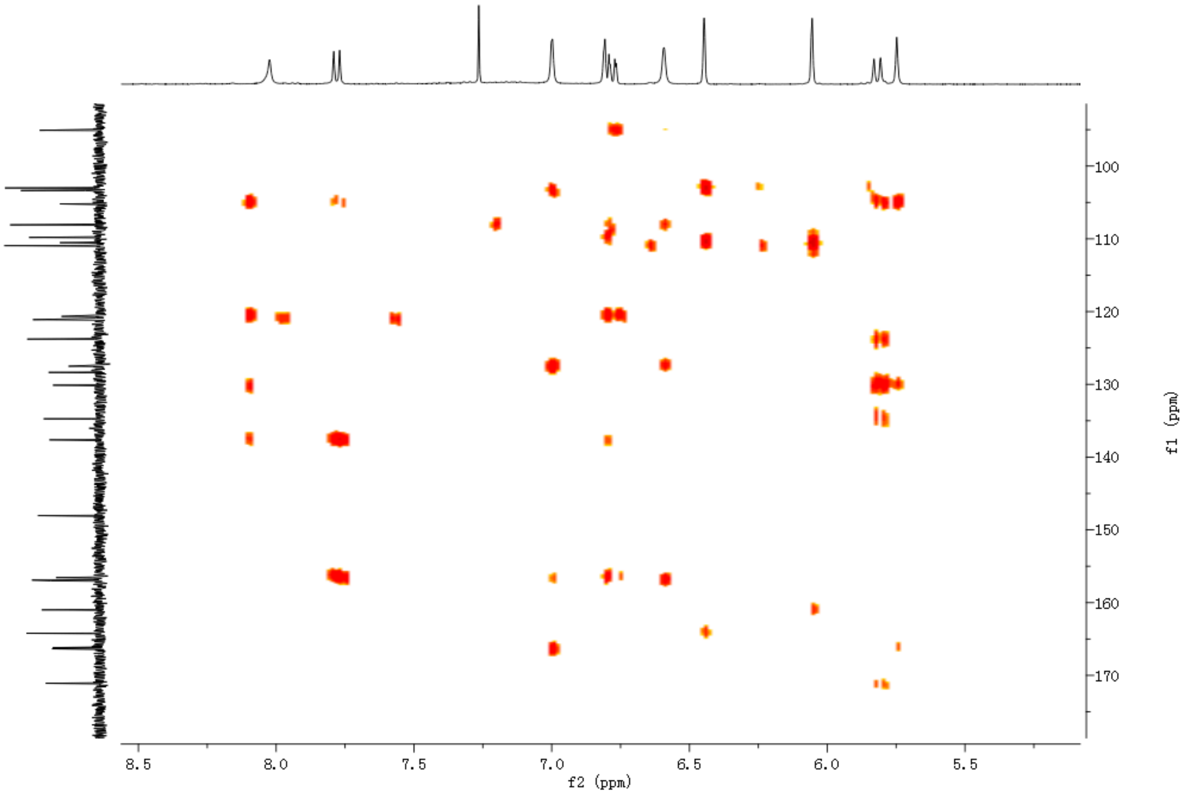

Figure S6. Expanded HMBC spectrum of compound **1**.

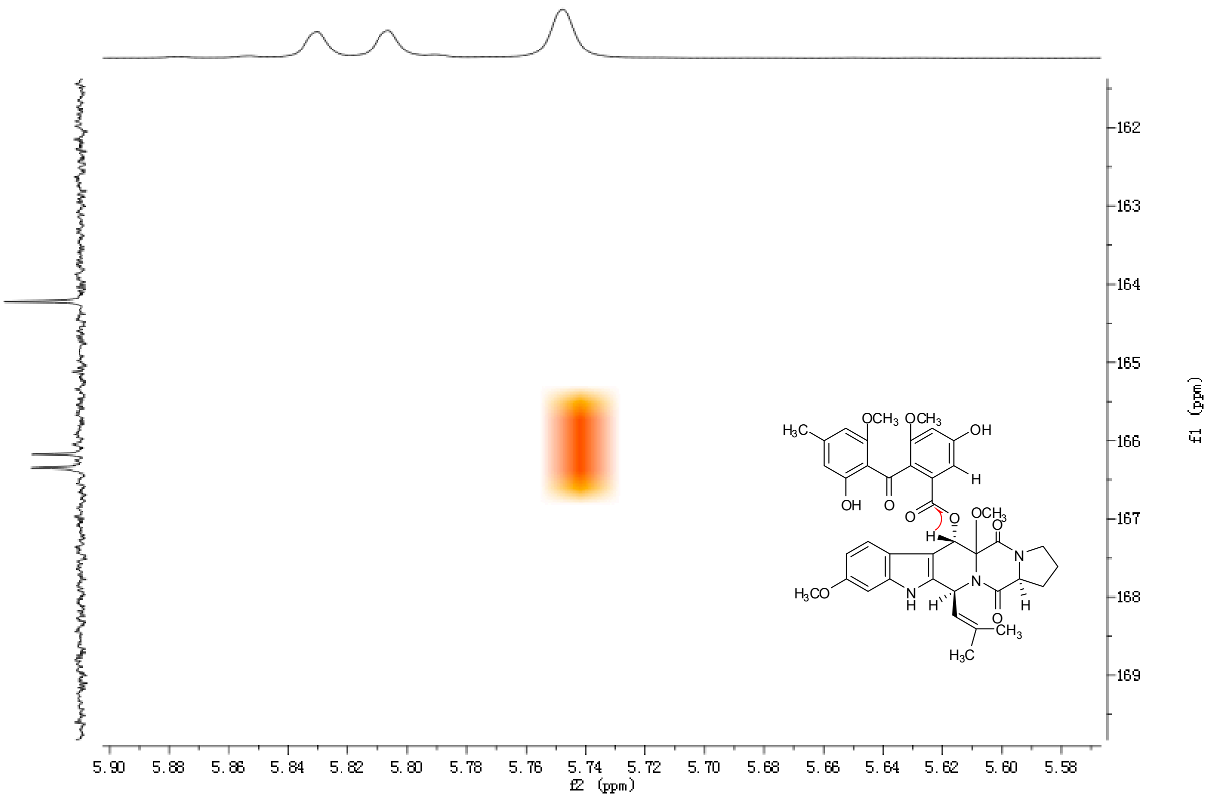

Figure S7. Key correlation signal of HMBC Spectrum of compound **1**.

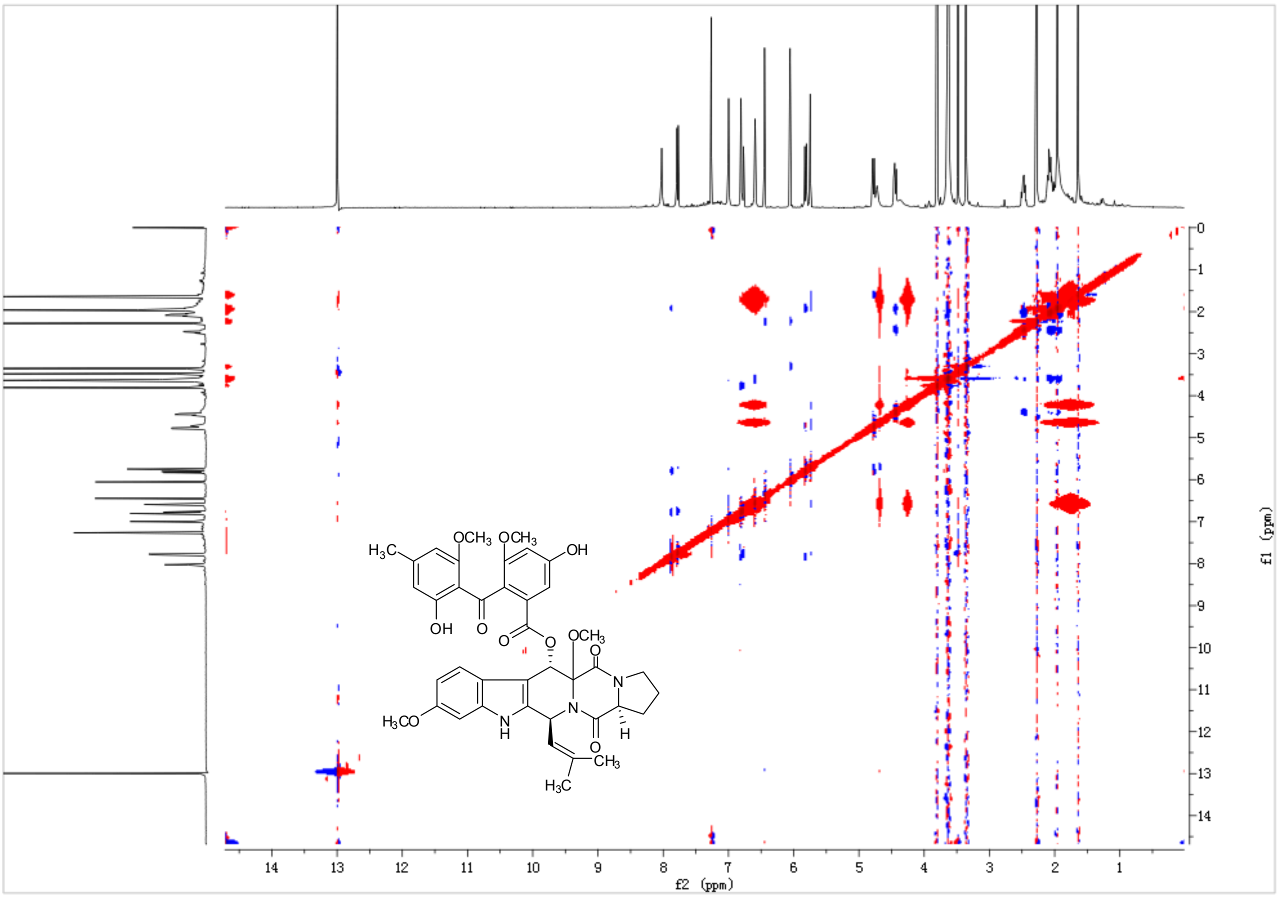

Figure S8. NOESY spectrum of compound **1**.

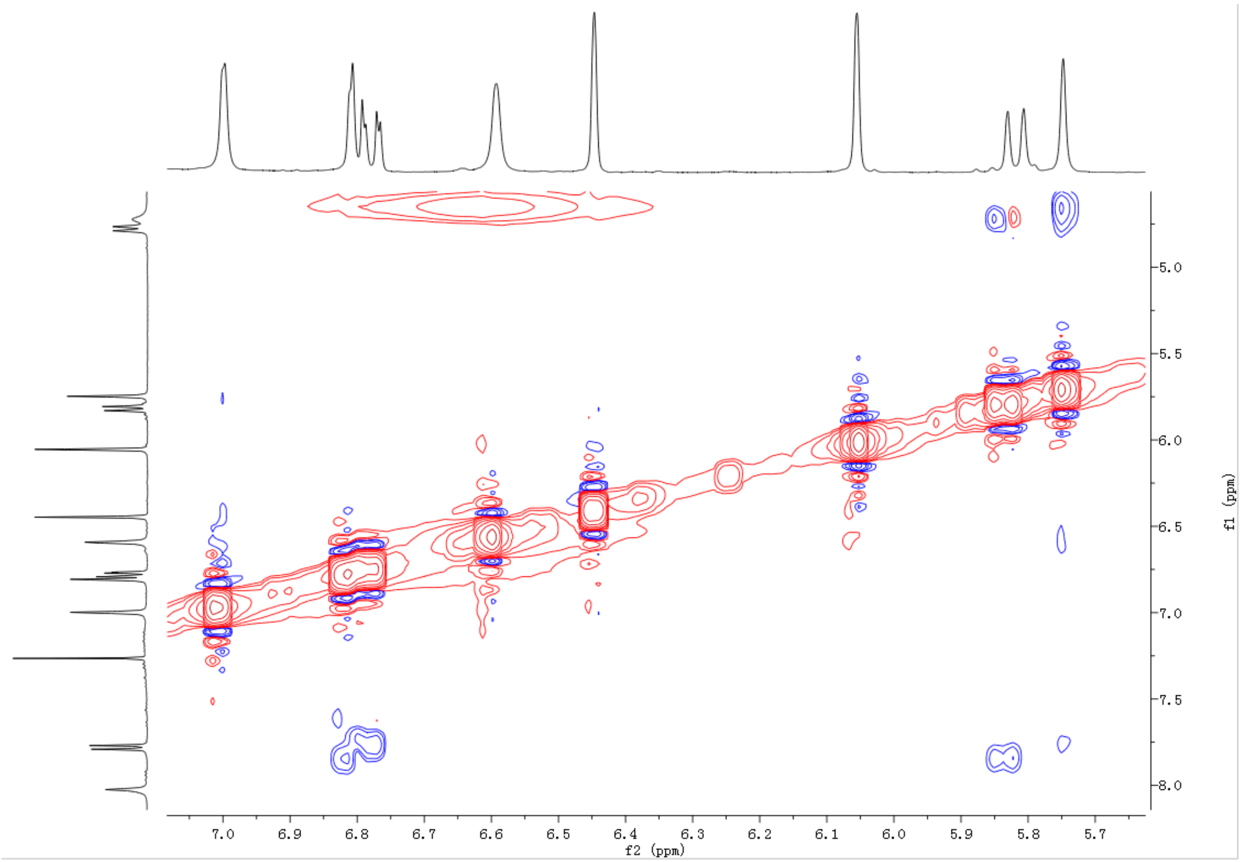

Figure S9. Expanded NOESY spectrum of compound **1**.

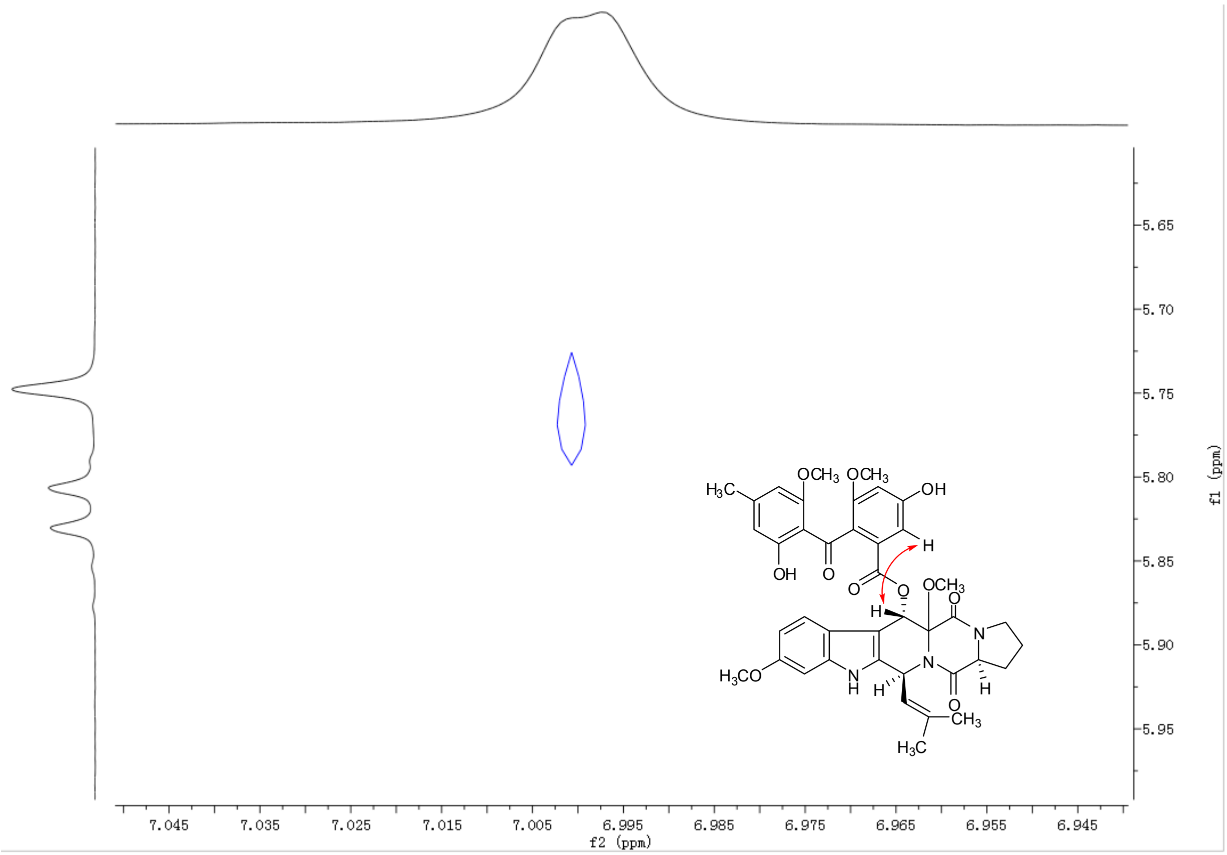

Figure S10. Key NOESY spectrum of compound **1**.

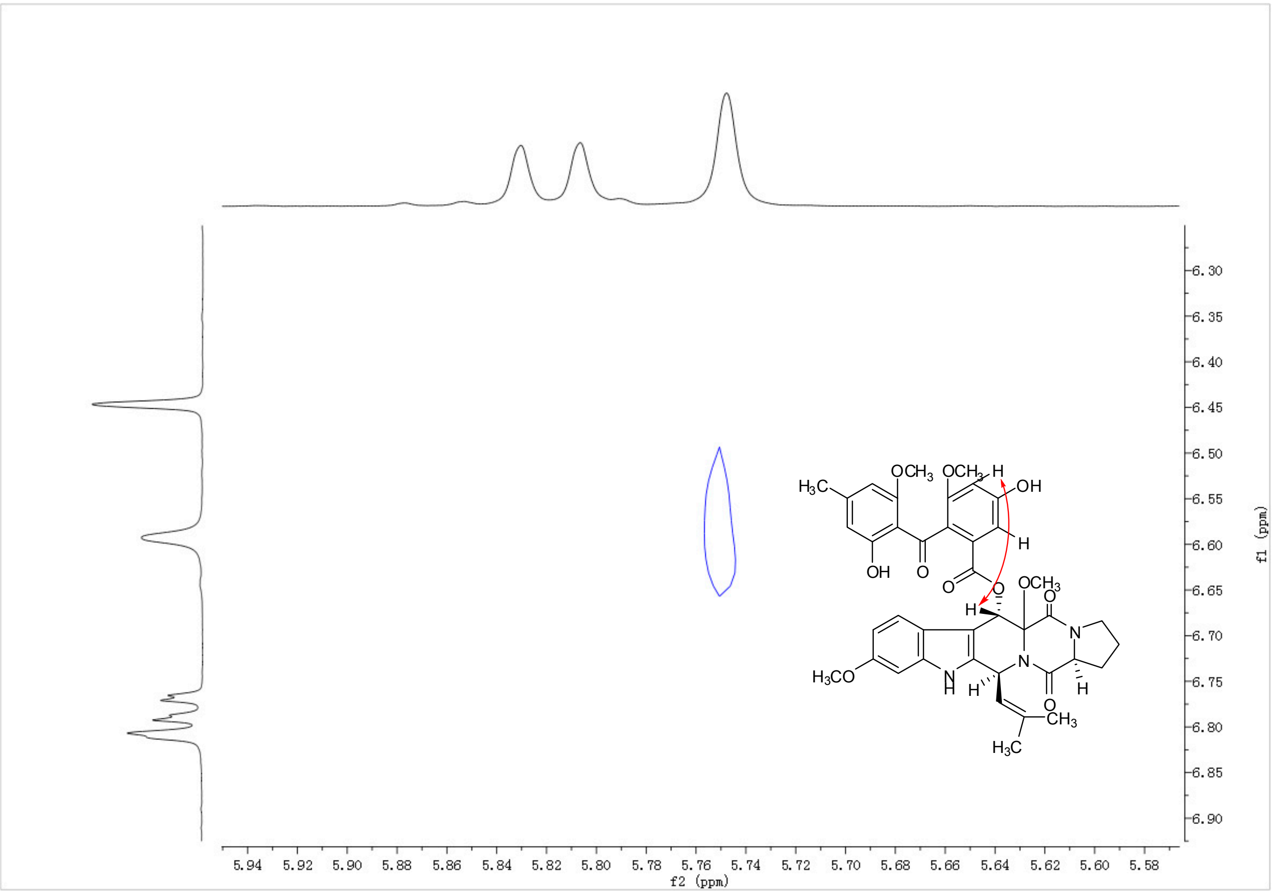

Figure S11. Key NOESY spectrum of compound **1**.

Acquisition Parameter

Source Type	ESI	Ion Polarity	Positive	Set Nebulizer	0.4 Bar
Focus	Active	Set Capillary	4500 V	Set Dry Heater	180 °C
Scan Begin	50 m/z	Set End Plate Offset	-500 V	Set Dry Gas	4.0 l/min
Scan End	1000 m/z	Set Collision Cell RF	150.0 Vpp	Set Divert Valve	Source

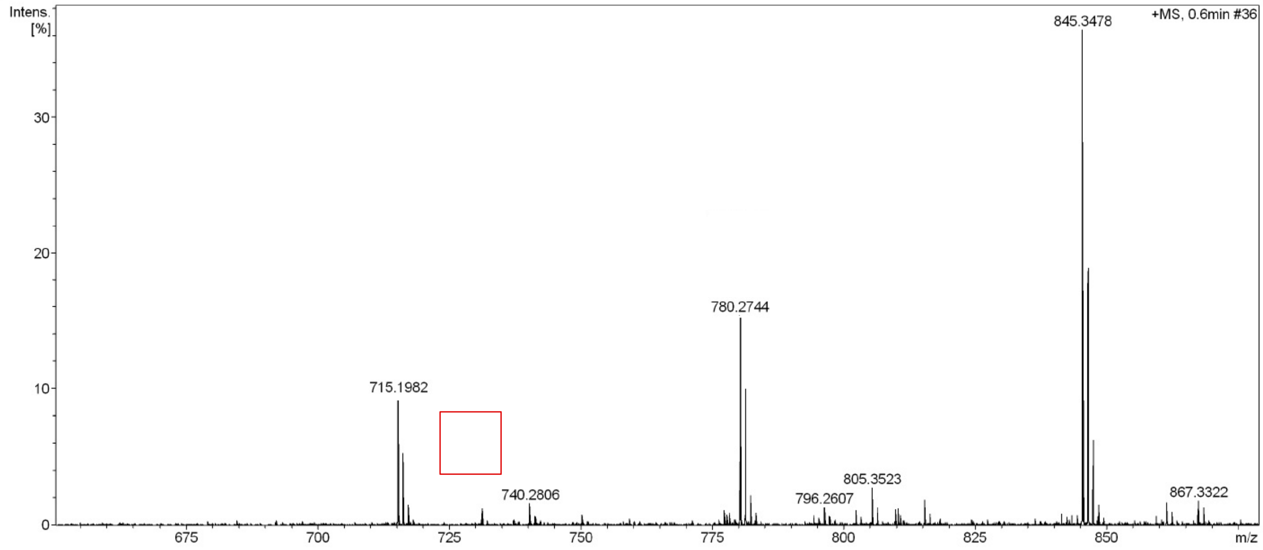

Meas. m/z	#	Formula	Score	m/z	err [mDa]	err [ppm]	mSigma	rdb	e⁻ Conf	N-Rule
740.2806	1	C 40 H 42 N 3 O 11	100.00	740.2814	0.8	1.1	68.7	21.5	even	ok

Bruker Compass DataAnalysis 4.0 Page 1 of 1

Figure S12. HR-ESIMS of compound **1**.

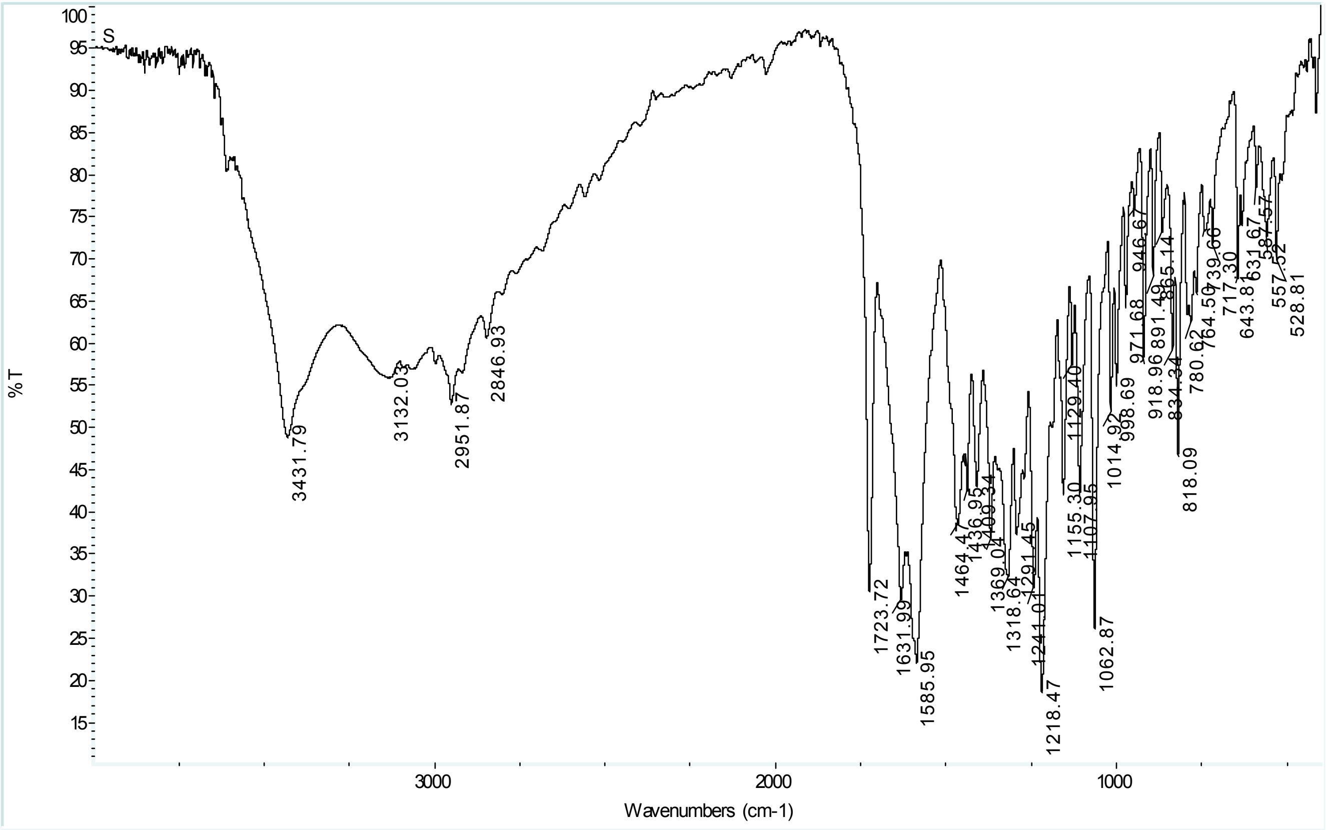

Figure S13. IR spectrum of compound **1**.

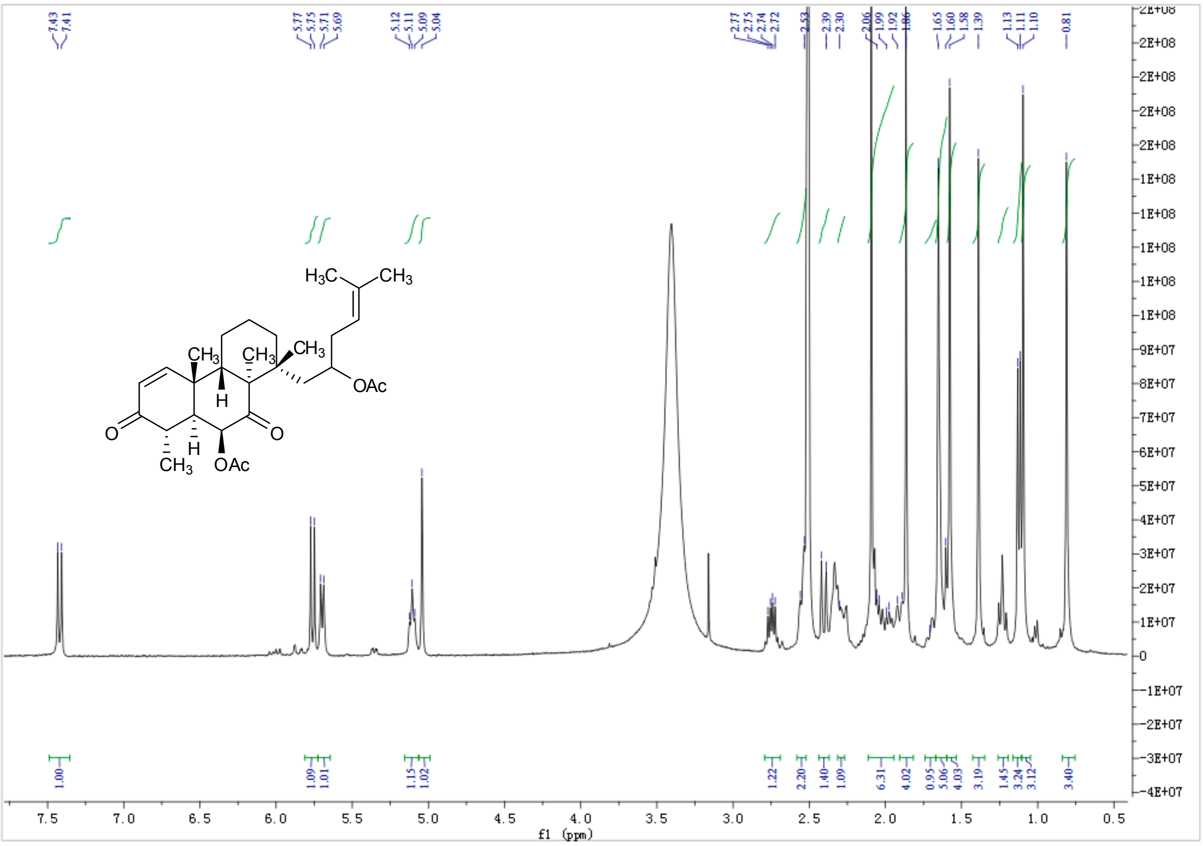

Figure S14. ^{1}H-NMR (400 MHz, DMSO-d_6) spectrum of compound **2**.

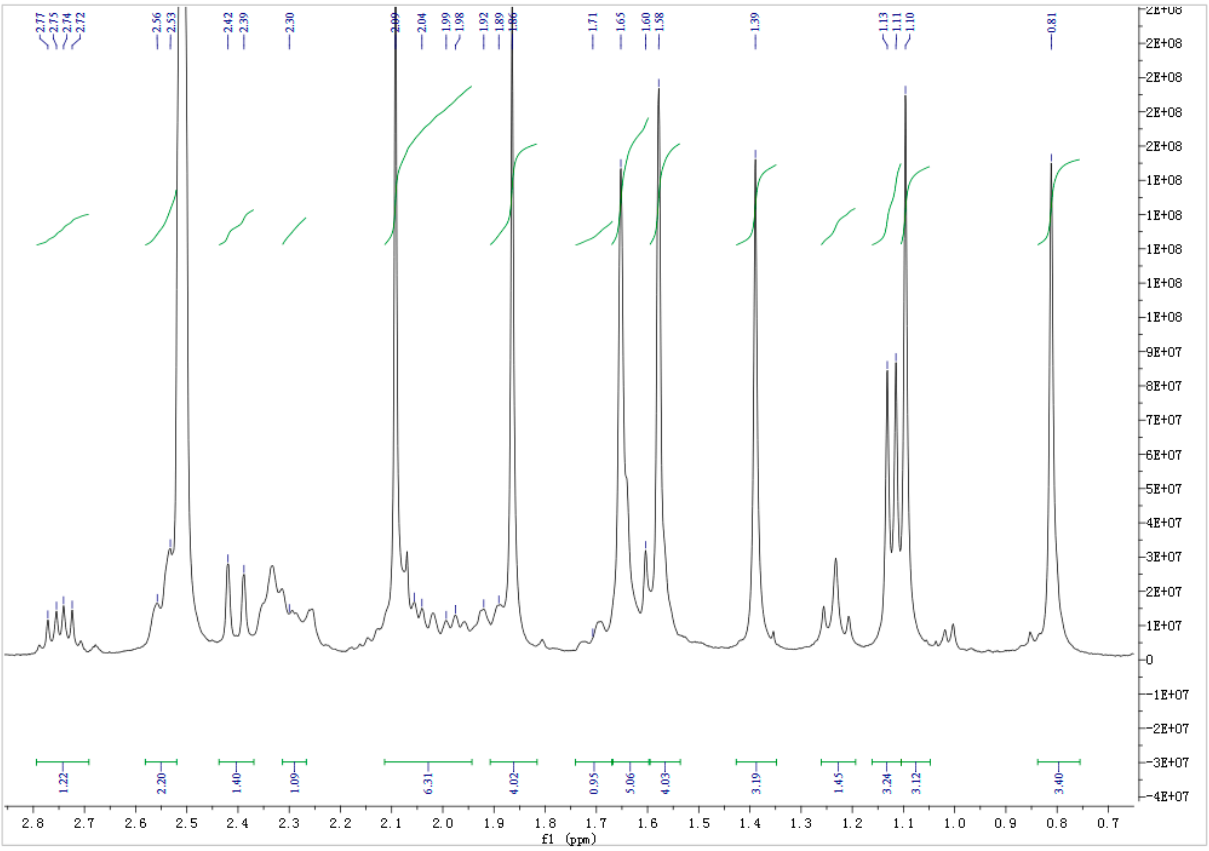

Figure S15. Expanded ^{1}H-NMR (400 MHz, DMSO-*d*6) spectrum of compound **2**.

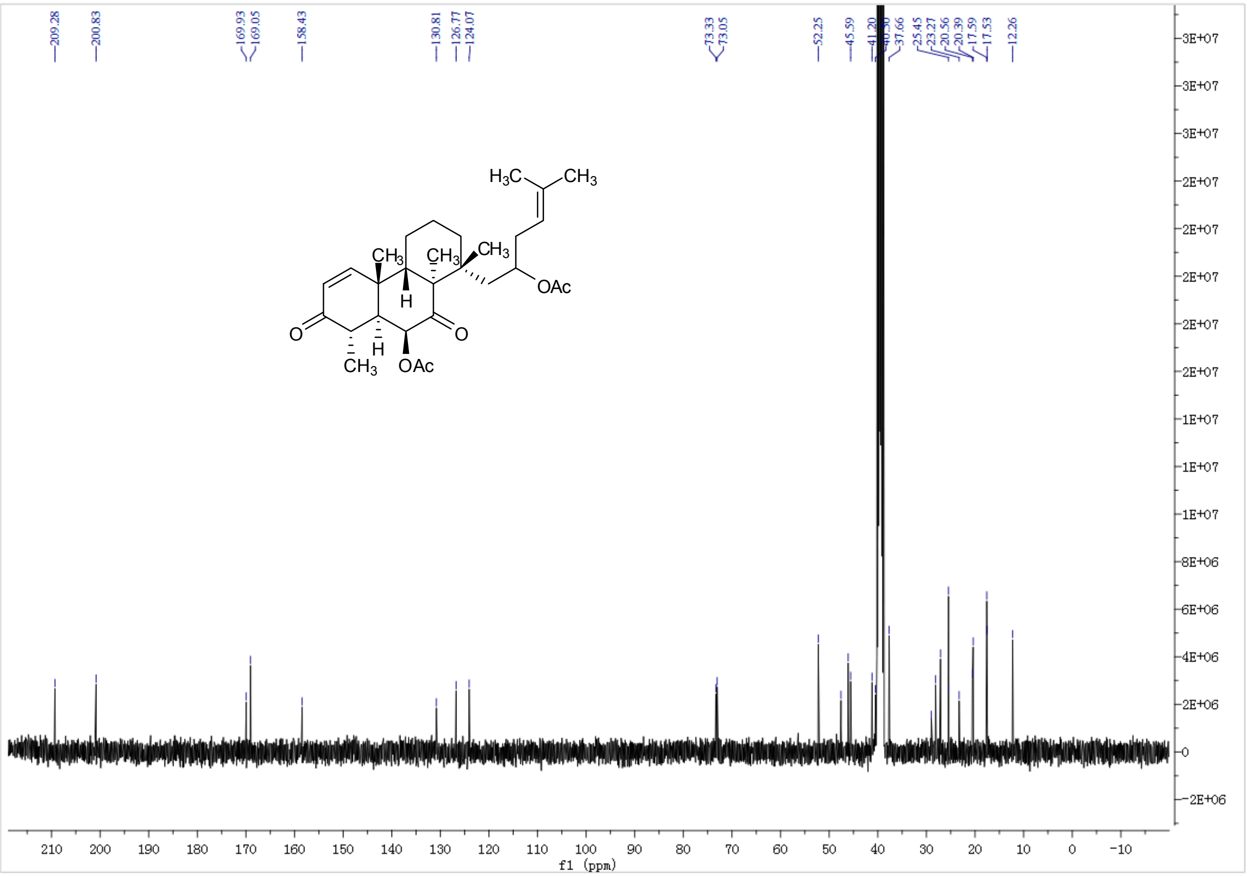

Figure S16. ^{13}C-NMR (100 MHz, DMSO-*d*6) Spectrum of compound **2**.

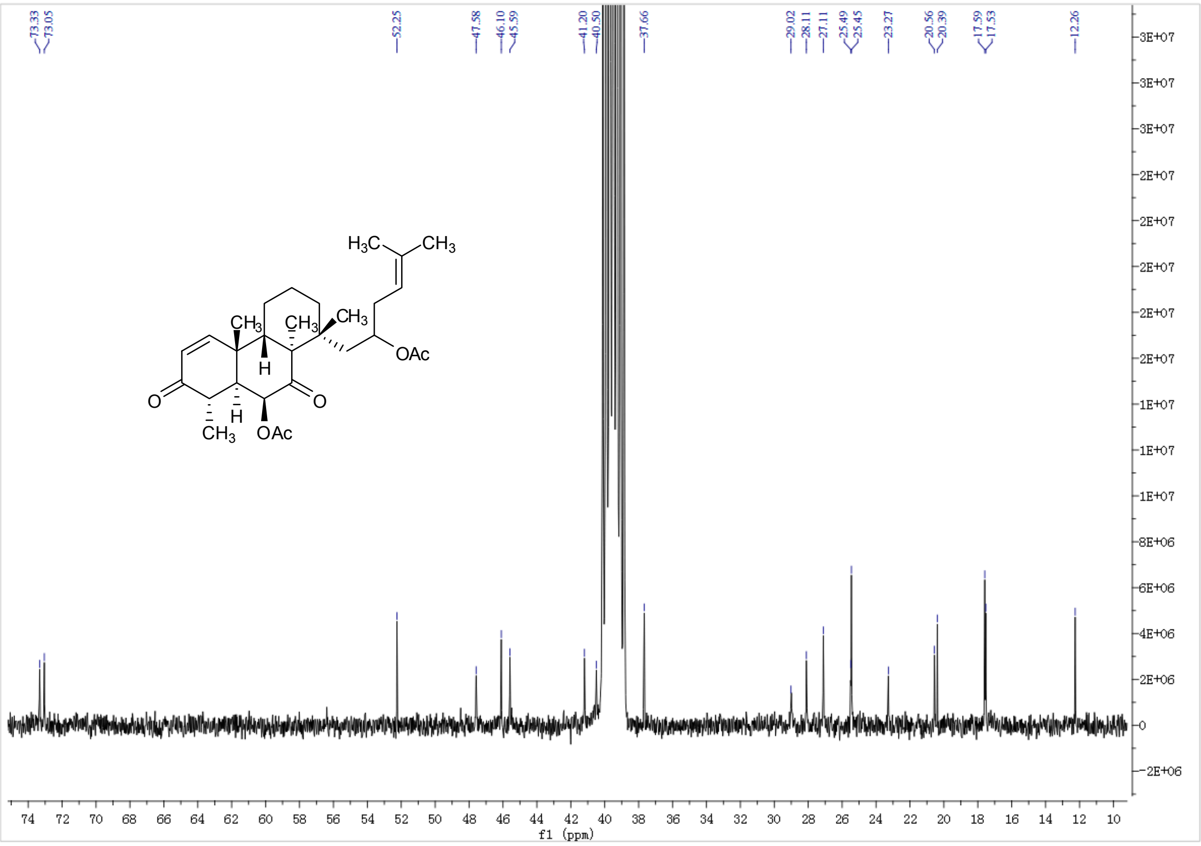

Figure S17. Expanded ^{13}C-NMR (100 MHz, DMSO-d_6) Spectrum of compound **2**.

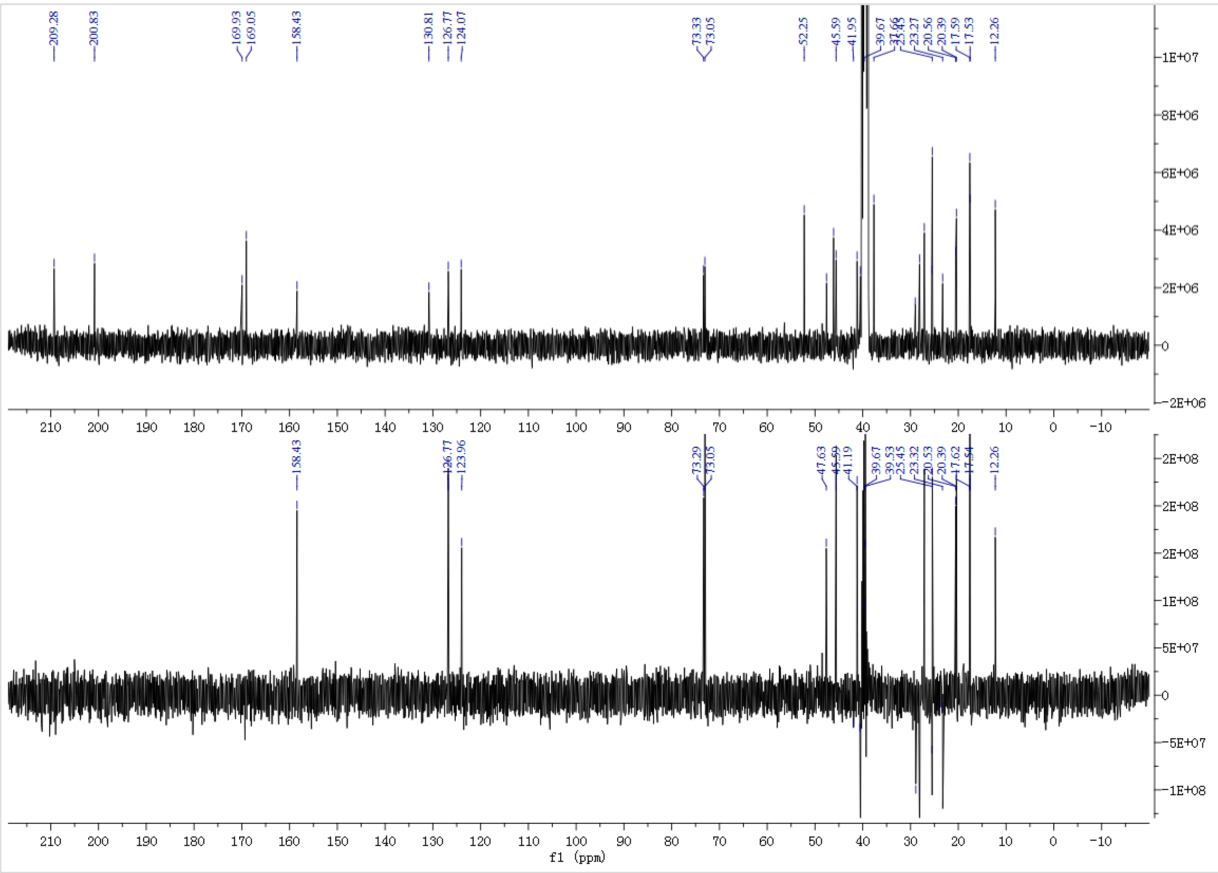

Figure S18. DEPT135 Spectrum (100 MHz, DMSO-d_6) of compound **2**.

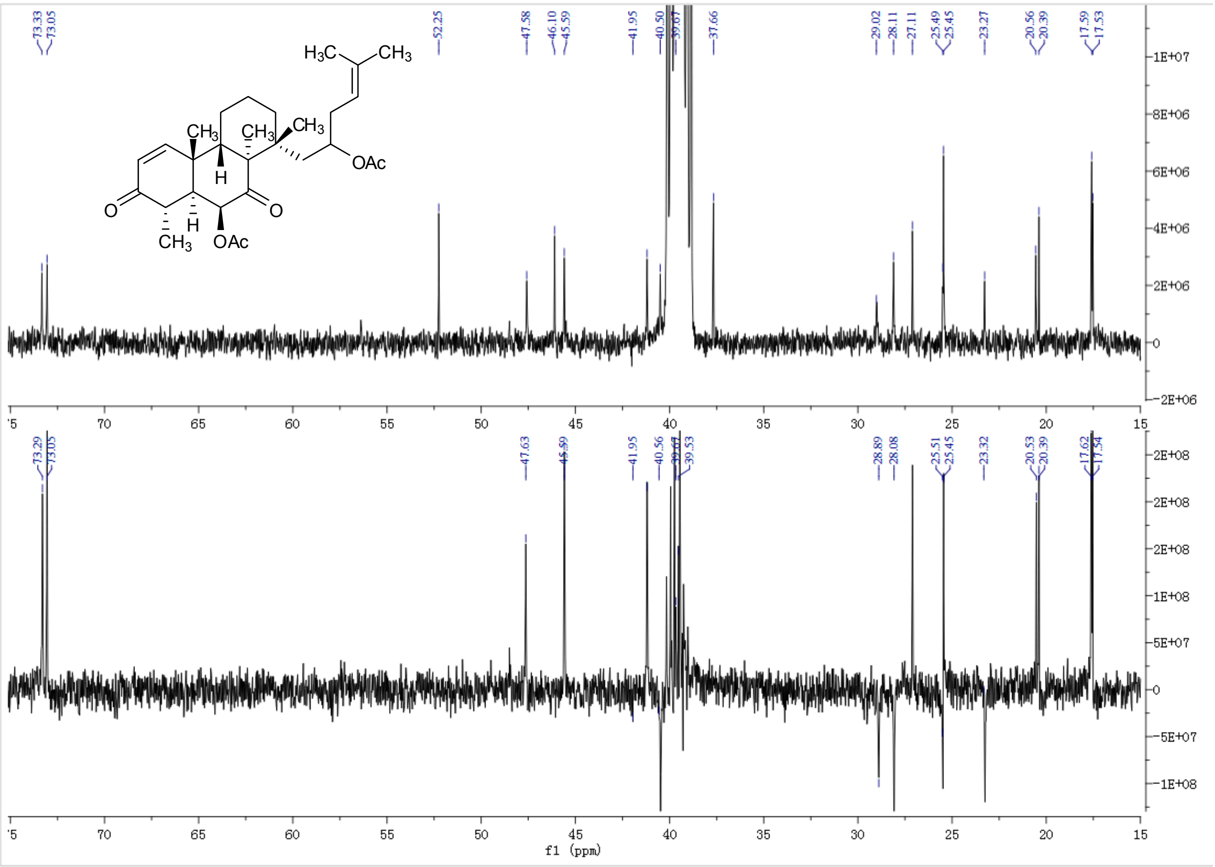

Figure S19. Expanded DEPT135 Spectrum (100 MHz, DMSO-d_6) of compound **2**.

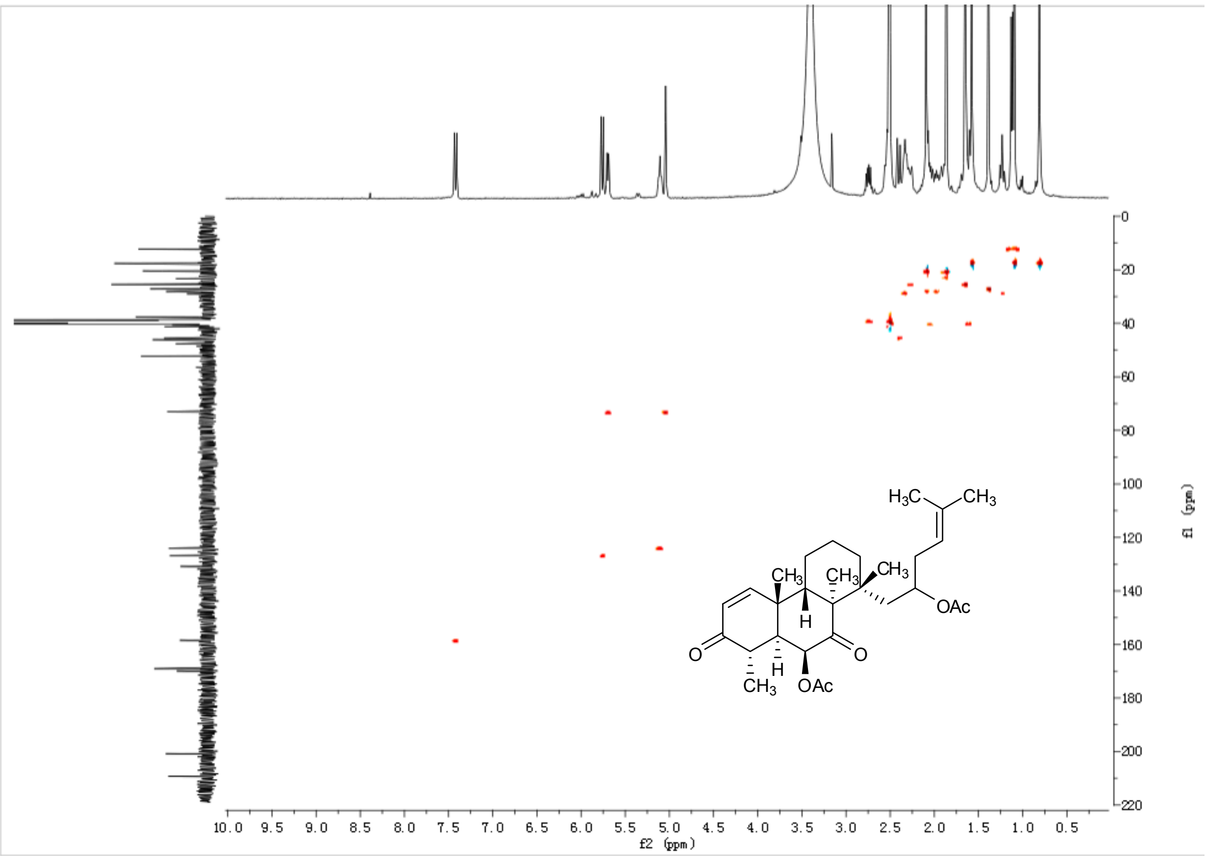

Figure S20. HSQC Spectrum of compound **2**.

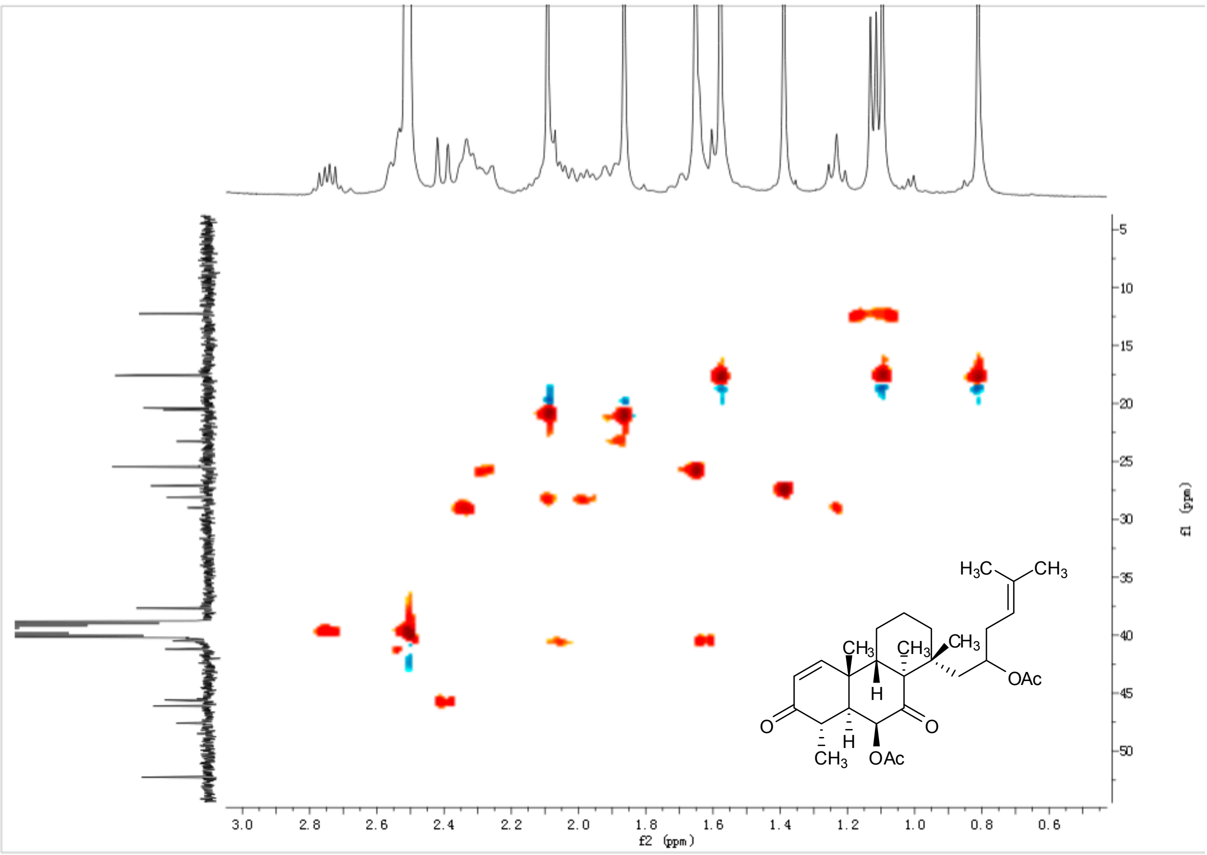

Figure S21. Expanded HSQC Spectrum of compound **2**.

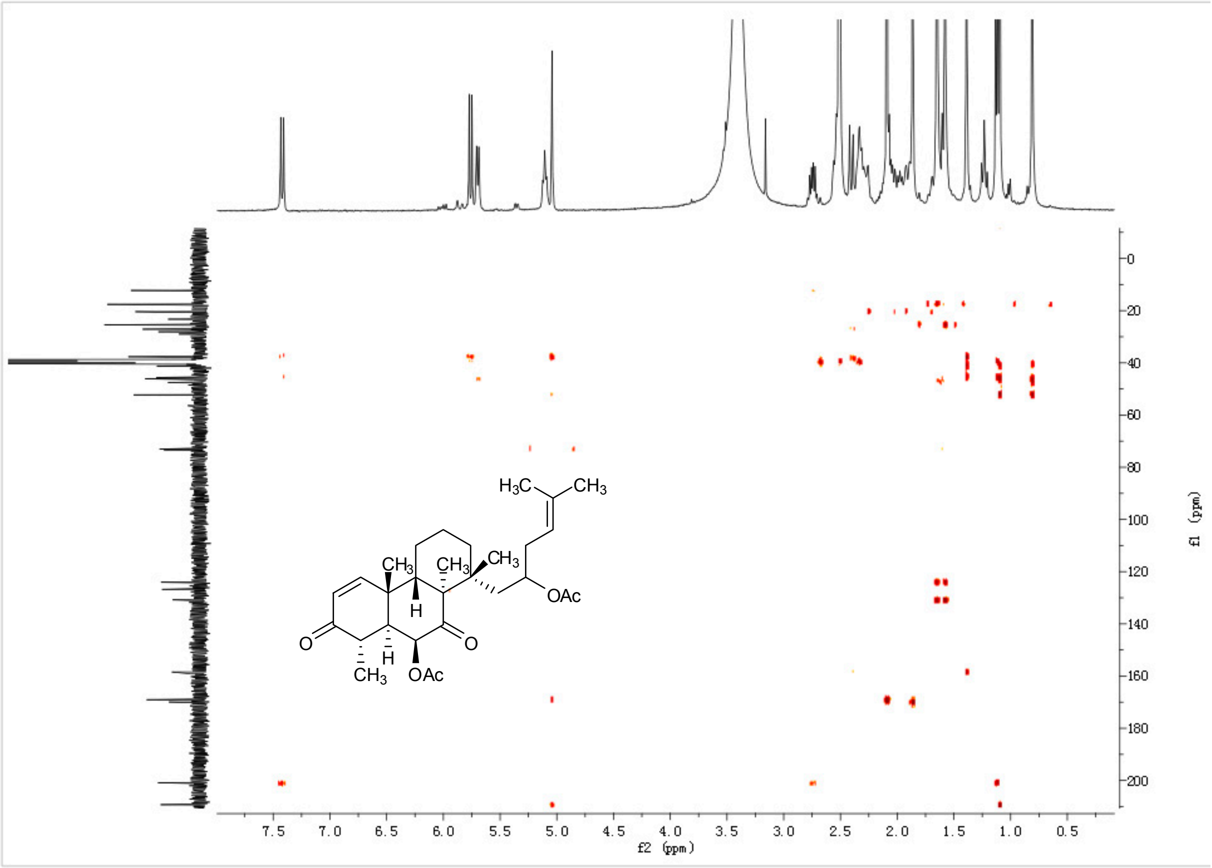

Figure S22. HMBC Spectrum of compound **2**.

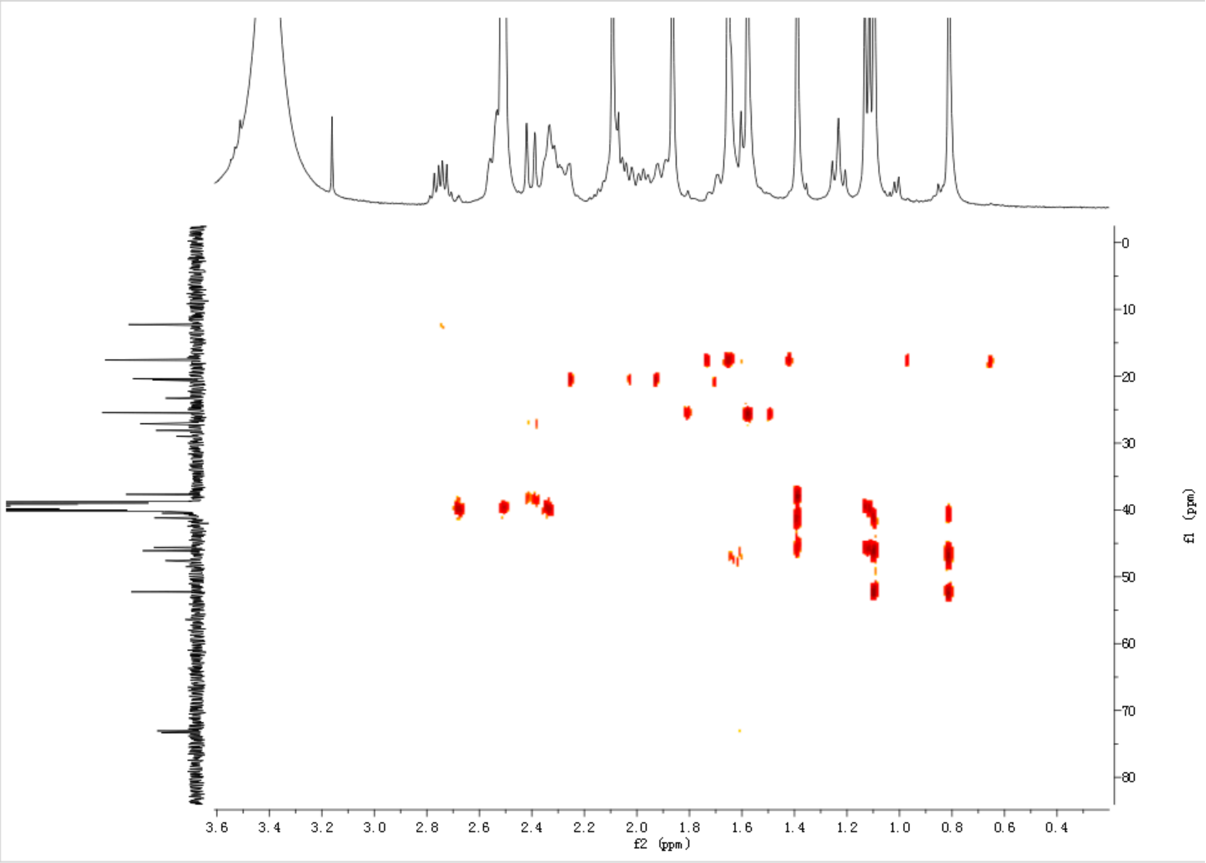

Figure S23. Expanded HMBC Spectrum of compound **2**.

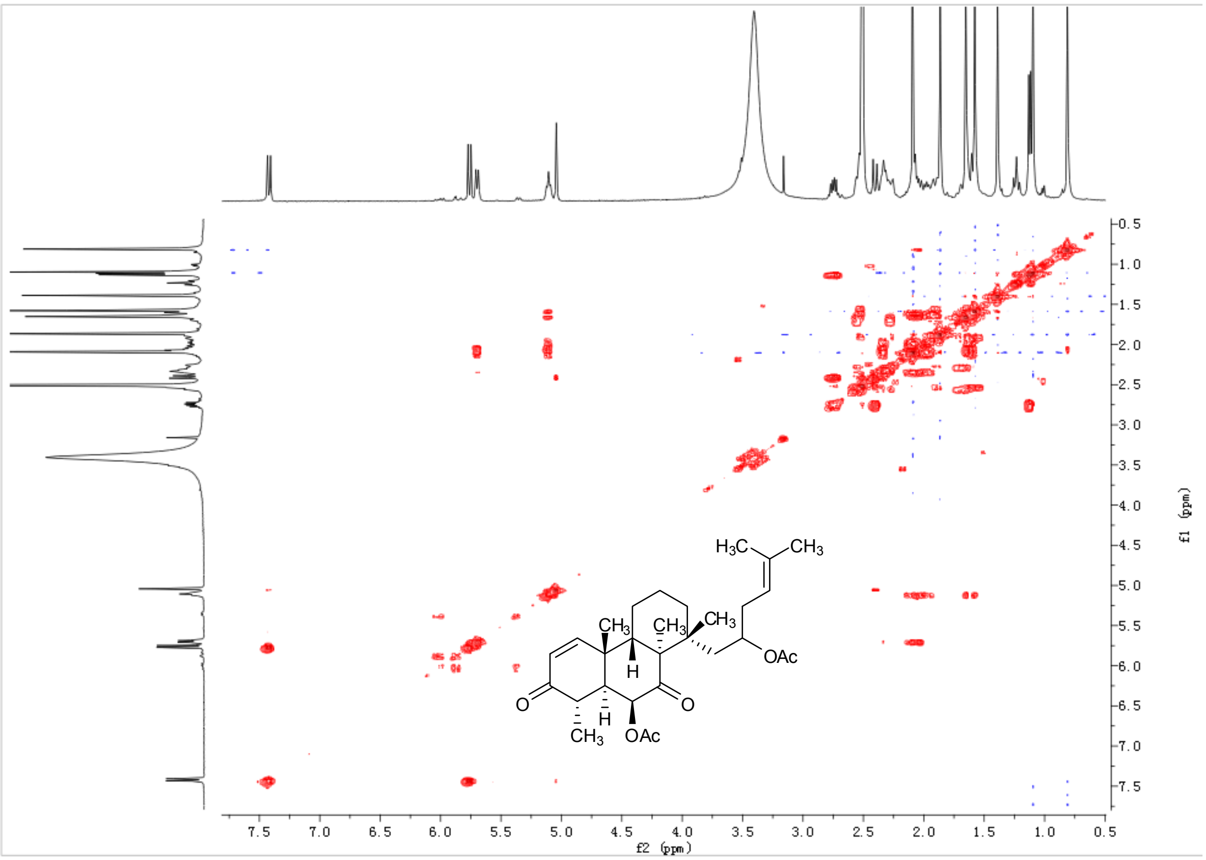

Figure S24. COSY Spectrum of compound **2**.

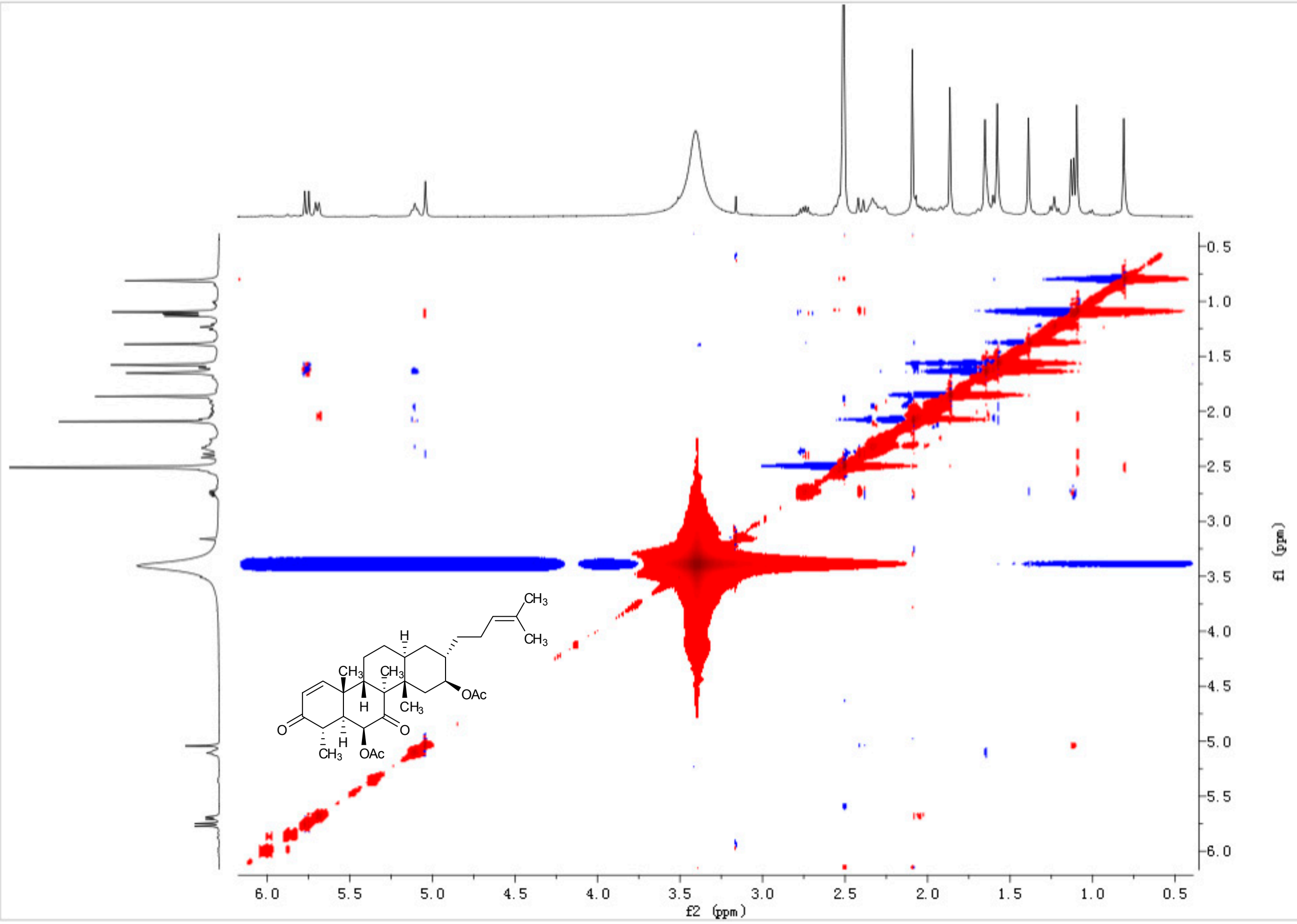

Figure S25. NOESY Spectrum of compound **2**.

Acquisition Parameter

Source Type	ESI	Ion Polarity	Positive	Set Nebulizer	0.4 Bar
Focus	Active	Set Capillary	4500 V	Set Dry Heater	180 °C
Scan Begin	50 m/z	Set End Plate Offset	-500 V	Set Dry Gas	4.0 l/min
Scan End	1000 m/z	Set Collision Cell RF	150.0 Vpp	Set Divert Valve	Source

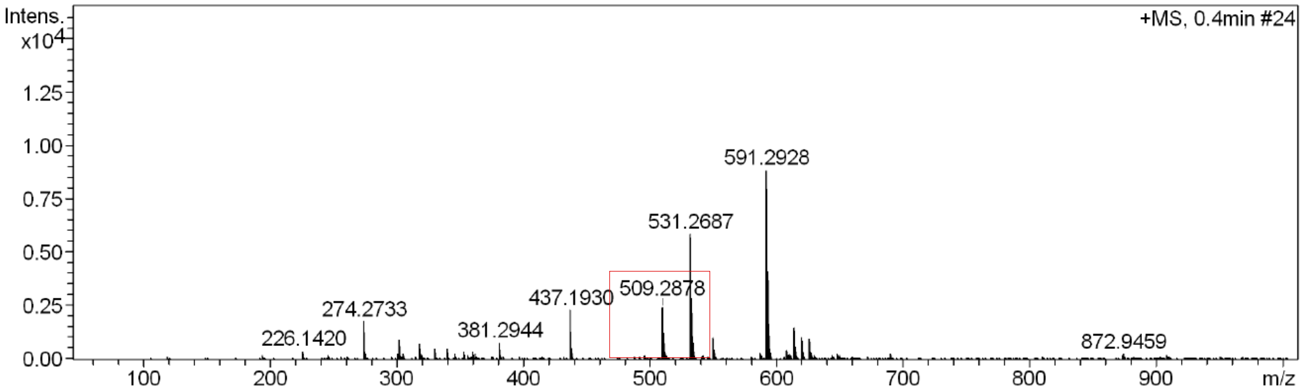

Meas. m/z	#	Formula	Score	m/z	err [mDa]	err [ppm]	mSigma	rdb	e⁻ Conf	N-Rule
509.2878	1	C 29 H 42 Na O 6	100.00	509.2874	-0.4	-0.8	33.6	8.5	even	ok

Figure S26. HR-ESIMS of compound **2**.
